# Catalysis with cycloruthenated complexes†

**DOI:** 10.1039/d1sc06355c

**Published:** 2022-02-17

**Authors:** Michael T. Findlay, Pablo Domingo-Legarda, Gillian McArthur, Andy Yen, Igor Larrosa

**Affiliations:** School of Chemistry, University of Manchester Oxford Road Manchester M13 9PL UK igor.larrosa@manchester.ac.uk

## Abstract

Cycloruthenated complexes have been studied extensively over the last few decades. Many accounts of their synthesis, characterisation, and catalytic activity in a wide variety of transformations have been reported to date. Compared with their non-cyclometallated analogues, cycloruthenated complexes may display enhanced catalytic activities in known transformations or possess entirely new reactivity. In other instances, these complexes can be chiral, and capable of catalysing stereoselective reactions. In this review, we aim to highlight the catalytic applications of cycloruthenated complexes in organic synthesis, emphasising the recent advancements in this field.

## Abstract 2

We discuss recent advances in the applications of cycloruthenated complexes in organic synthesis, comprising C–H activation, chiral-at-metal catalysis, *Z*-selective olefin metathesis, transfer hydrogenation, enantioselective cyclopropanations and cycloadditions.

## Graphical abstract

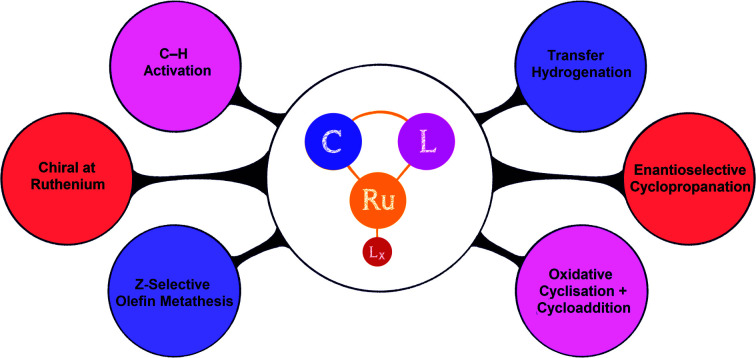

## 1. Introduction

The discovery and development of novel and efficient catalysts that can facilitate new chemical transformations has been a longstanding challenge in chemical synthesis. Significant effort has been directed towards the development of novel transition-metal complexes for metal-catalysed organic synthesis. The assembly of well-defined complexes from metal and ligand combinations enables fine-tuning of catalytic properties, making metal complexes an attractive option for modular catalyst design.^1^

Cyclometallation of transition-metal complexes is the simplest way of forming a metal–carbon bond. Consequently, this method has been widely studied and applied towards the synthesis of cyclometallated complexes.^2^ The first examples of these complexes were reported in the 1960s, and since then, there have been numerous reports detailing their synthesis, properties and reactivity.^3^

Cyclopalladated complexes such as the Hermann–Beller palladacycle and Buchwald G2 precatalyst are noteworthy examples of cyclometallated complexes that are extensively applied in organic synthesis.^4^ In contrast, ruthenium has been relatively underexplored, but is becoming increasingly popular in the search for new and complementary modes of reactivity.

Ruthenium offers several benefits that make it an attractive choice for use in organic synthesis. Compared to other transition metals (*e.g.* palladium, platinum, and rhodium), ruthenium is inexpensive, making its use in both academic and industrial settings more economically viable.^5^ Ruthenium complexes are often straightforward to synthesise and characterise due to their stability under ambient conditions. In addition, ruthenium complexes display a diverse range of reactivity.

This review seeks to highlight catalytic bond-forming applications of cycloruthenated complexes that are relevant to organic synthesis. Reports that provide strong evidence for the involvement of cycloruthenated intermediates or those which utilise cycloruthenated precatalysts will be emphasised.

Accordingly, this review will be organised by reaction type, comprising six areas in which cycloruthenated complexes have been most impactful: C–H activation, chiral-at-metal catalysis, *Z*-selective olefin metathesis, transfer hydrogenation, enantioselective cyclopropanations, oxidative cyclisation and cycloadditions. While dedicated reviews on each of these topics have been published,^6–11^ this review will be focusing on the advantages and breadth of reactivity offered by cycloruthenated complexes. These advantages will be emphasised to the reader where applicable. Significant advances in the chemistry of cycloruthenated complexes since Pfeffer's 2009 review necessitates an update of this field.^12^ As the focus will be on catalytic bond-forming applications, we will not discuss cycloruthenated complexes in the context of DSSCs (dye-sensitised solar cells), water oxidation, or polymerisation reactions.

It is our hope that this review will draw attention to the unique and useful behaviour of cycloruthenated species in catalysis. We hope that by highlighting the recent advances and existing challenges in this field, this review can encourage further research into the development of useful new catalysts for organic synthesis.

## 2. C–H functionalisation

Transition metal catalysed C–H functionalisation is invaluable for the direct transformation of ubiquitous C–H bonds into valuable C–X bonds (typically X = B, C, N, or O), facilitating the rapid construction of molecular complexity. In general, ruthenium-catalysed C–H functionalisation leads to the formation of cyclometallated ruthenium intermediates, many of which have been isolated and characterised. Although these species have been reported, the exact nature and role of these species in catalytic processes are scarcely investigated in detail.

Given that cycloruthenated complexes are ubiquitous in directed C–H functionalisation, an exhaustive list of every reaction that implicates a cycloruthenated intermediate is beyond the scope of this review. Instead, this section will focus on the advances in C–H functionalisation achieved with the use of discrete cyclometallated ruthenium species, and studies where cyclometallated intermediates have been isolated and/or characterised. This section comprises four parts: alkylation; arylation; sulfonation, nitration, and acylation; and annulation reactions.

### 2.1 Alkylation

**Scheme 1:**
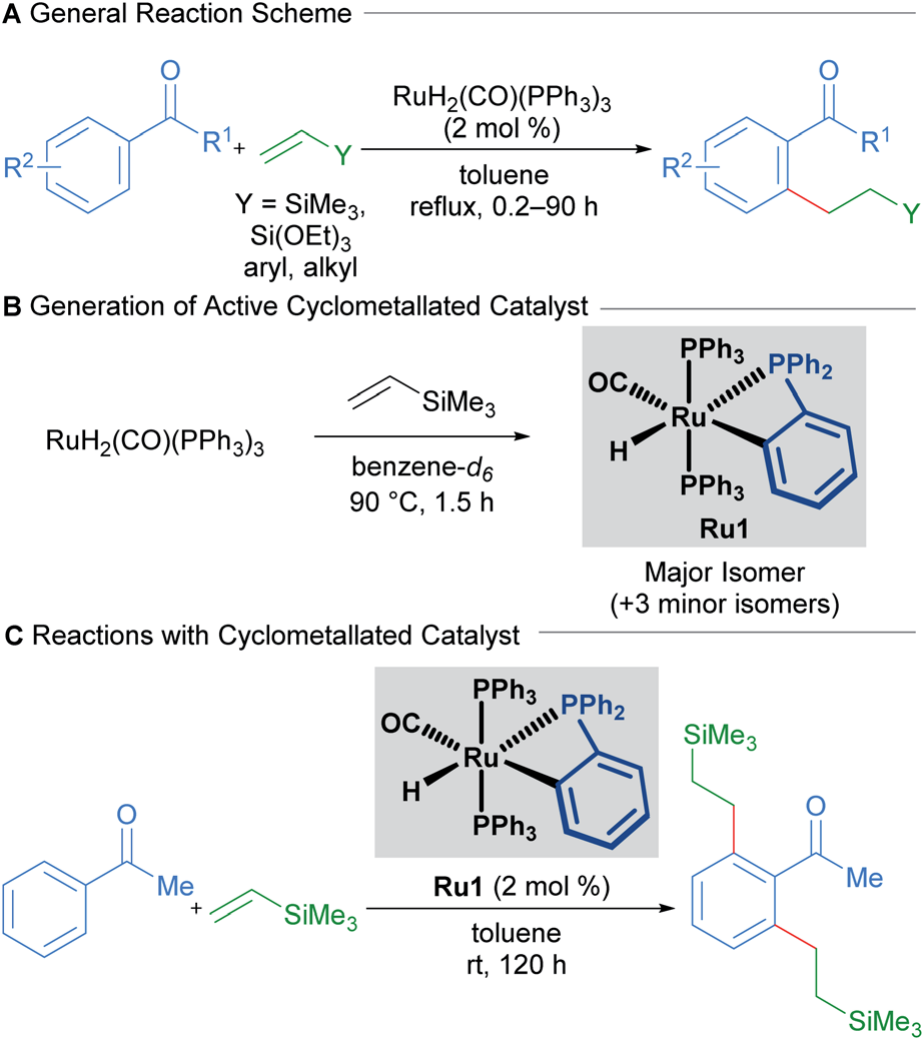
Room-temperature C–H/olefin coupling reported by Murai.

Transition-metal-catalysed C–H alkylation reactions typically involve the transformation of C(sp^2^)–H bonds into more valuable C(sp^2^)–C(sp^3^) bonds. In a seminal paper published in 1993, Murai describes the ruthenium-catalysed directed C–H alkylation of arylketones utilising a range of alkenes to furnish *ortho*-functionalised products (Scheme 1A).^13^

The proposed mechanism invokes a cyclometallated ruthenium intermediate to account for the high regioselectivity of this transformation. Since then, the groups of Murai and others have reported various ruthenium-catalysed alkylation procedures with alkenes that invoke similar mechanisms.

In 2010, a mechanistic investigation by the Murai group uncovered a more reactive catalyst (Ru1) for the regioselective coupling between aromatic ketones and olefins.^14^ The authors found that the high temperatures required for the reaction using RuH_2_(CO)(PPh_3_)_3_ as a pre-catalyst were only required for the formation of the cyclometallated active catalytic species (Scheme 1B). Consequently, prior generation by heating RuH_2_(CO)(PPh_3_)_3_ in the presence of trimethylvinylsilane allowed the reaction to proceed at room-temperature (Scheme 1C). The authors also found that using this new catalytic species enabled significantly lower catalyst loadings than before, with a TON of 994.

The Ackermann group has also published work on the development of ruthenium-catalysed alkylation procedures, utilising unactivated alkyl halides and a ruthenium complex as a pre-catalyst for the C–H alkylation of arenes that contain nitrogen-based heterocyclic directing groups. The regioselectivity of this transformation strongly depends on the type of alkyl halide employed; primary alkyl halides give exclusively the *ortho*-alkylation products, while secondary and tertiary alkyl halides lead to *meta*-alkylation products.

**Scheme 2:**
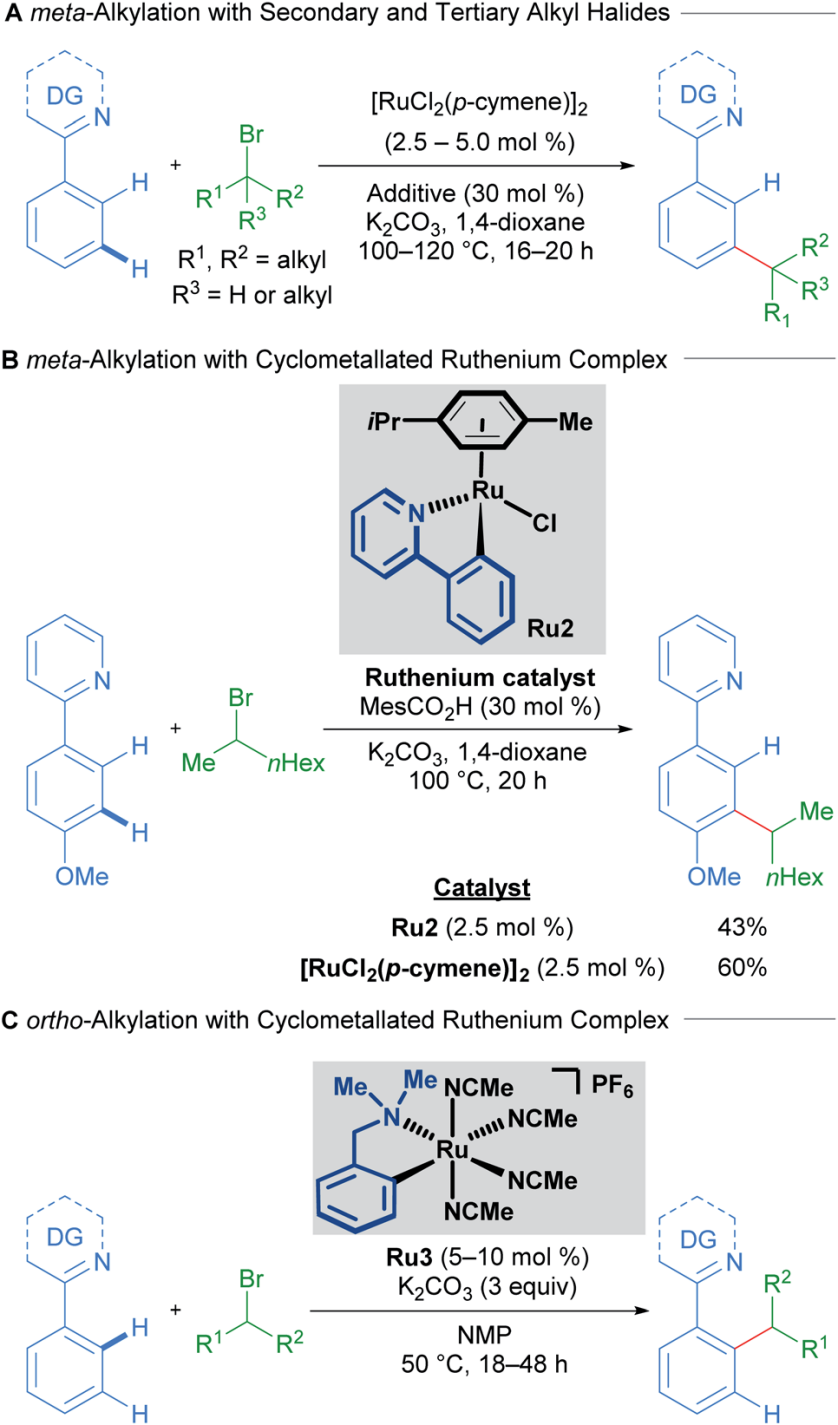
Selectivity switch with a cyclometallated ruthenium catalyst for C–H alkylation using secondary alkyl halides. DG = directing group.

The initial reports of ruthenium-catalysed C–H alkylation employing secondary and tertiary alkyl halides as electrophiles furnished the *meta*-alkylated products through a proposed σ-activation pathway (Scheme 2A).^15,16^ The authors showed that *p*-cymene-bound cycloruthenated complex Ru2 is a suitable precatalyst for *meta*-alkylation with both secondary (Scheme 2B) and tertiary alkyl bromides. Conversely, a recent study into C–H alkylation with secondary alkyl bromides by the Larrosa group demonstrates the complementary selectivity afforded by a novel *p*-cymene-free cyclometallated catalyst Ru3, delivering *ortho*-functionalised products instead of the usual *meta*-alkylation products at temperatures as low as 50 °C (Scheme 2C).^17^ Subsequently, Ackermann and coworkers showed that pyrazolylarene substrates can lead to both *ortho*- and *meta*-alkylated products at 120 °C depending on both the secondary alkyl bromide and the directing group used, even when using [RuCl_2_(*p*-cymene)]_2_ as catalyst, proposing the *in situ* formation of the *p*-cymene-free ruthenacycle.^18,19^

**Scheme 3:**
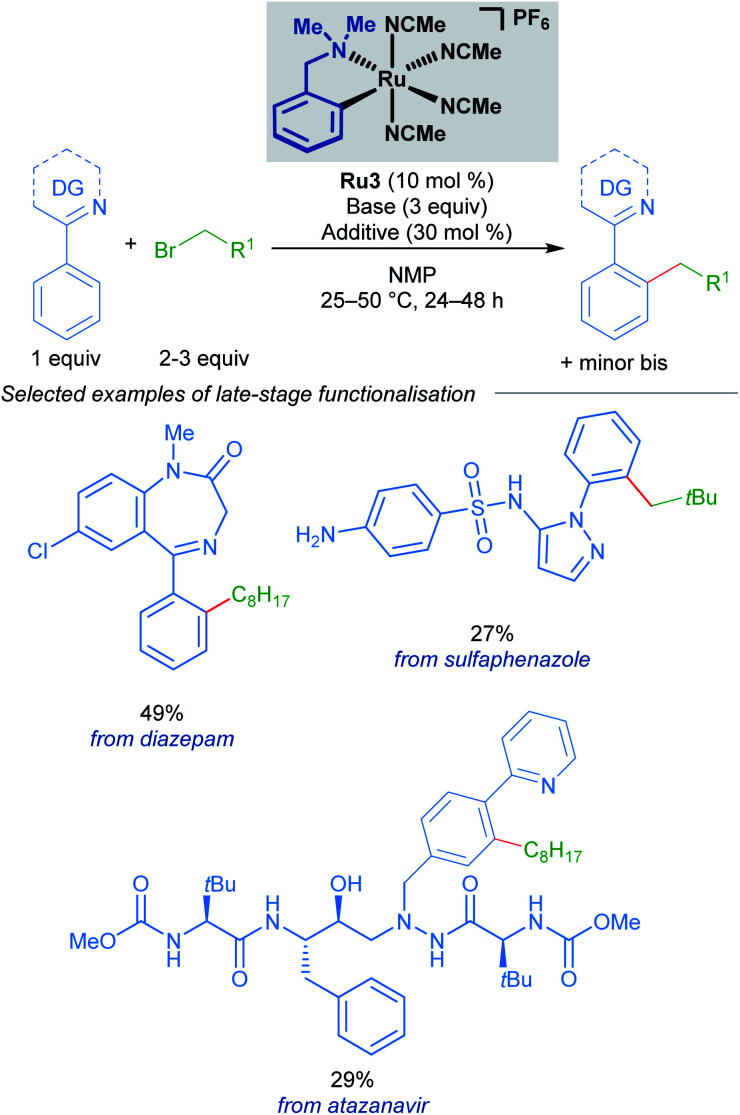
Larrosa's late-stage *ortho*-alkylation of N-directing group-containing arenes procedure using primary alkyl halides.

**Scheme 4:**
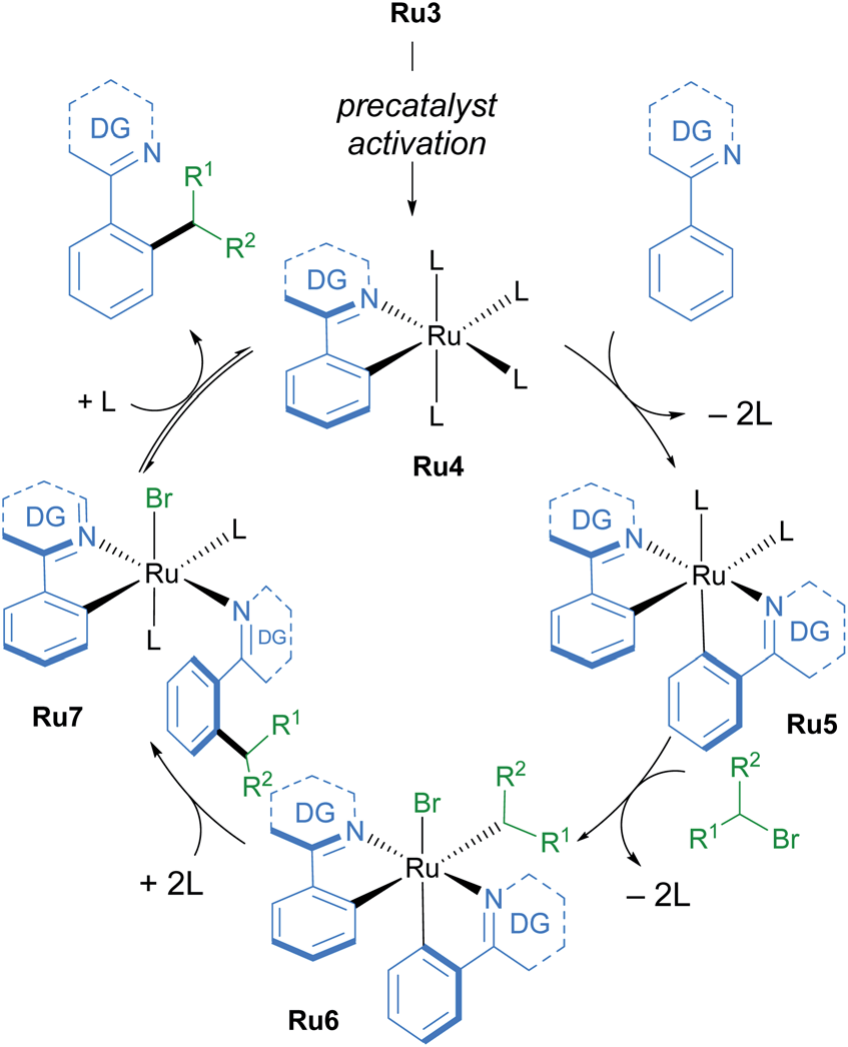
Mechanism of C–H functionalisation of N-directing group-containing arenes with primary and secondary alkyl bromides by Larrosa.

A follow-up study from the Larrosa group demonstrates that mono-cyclometallated complex Ru3 is also effective for *ortho*-alkylation using primary alkyl bromides.^20^ The use of this new catalyst allowed for very mild reaction conditions, expanding the scope of potential coupling partners, and permitting its use in late-stage functionalisation of complex molecules (Scheme 3). The proposed mechanism is supported by a combination of stoichiometric, kinetic, and *in situ* experiments (Scheme 4). Initial activation of the precatalyst Ru3 leads to the formation of an on-cycle monocyclometallated intermediate Ru4, and a second C–H activation event then forms the bis-cyclometallated complex Ru5. Contrary to previous reports on C–H alkylation, this bis-cyclometallated complex Ru5 is required to facilitate the oxidative addition of both primary and secondary alkyl bromides to the ruthenium centre. Notably, this intermediate is sufficiently electron-rich to promote oxidative addition at room temperature in the case of primary alkyl bromides, and at a moderate temperature for secondary alkyl bromides, to form Ru6. Subsequent reductive elimination affords intermediate Ru7, in which the final product is N-ligated to the ruthenium centre, and dissociation then yields the alkylated product and regenerates the active catalytic species.

### 2.2 Arylation

Oi and Inoue reported the first examples of ruthenium-catalysed *ortho*-arylation, utilising a [RuCl_2_(C_6_H_6_)]_2_ precatalyst.^21–23^ The authors show that both directed arylation and alkenylation of arenes was possible, demonstrating that pyridines, oxazolines, pyrazoles, as well as imines, are all suitable directing groups.

**Scheme 5:**
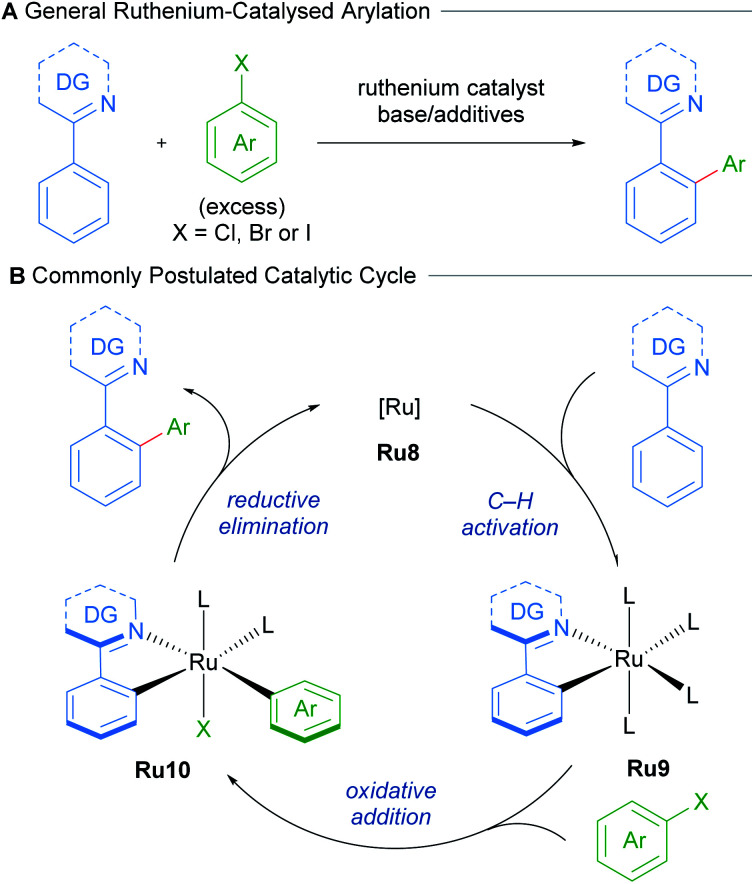
Commonly postulated mechanism for C–H arylation of N-directing group arenes with ruthenium.

Following this seminal report, further research has greatly expanded the scope of directing groups and coupling partners that can be employed in this reaction.^24^ These procedures utilise non-cyclometallated ruthenium precatalysts. These presumably undergo cyclometallation to afford mono-cyclometallated ruthenium intermediates that engage in subsequent oxidative addition and reductive elimination (Scheme 5).

**Scheme 6:**
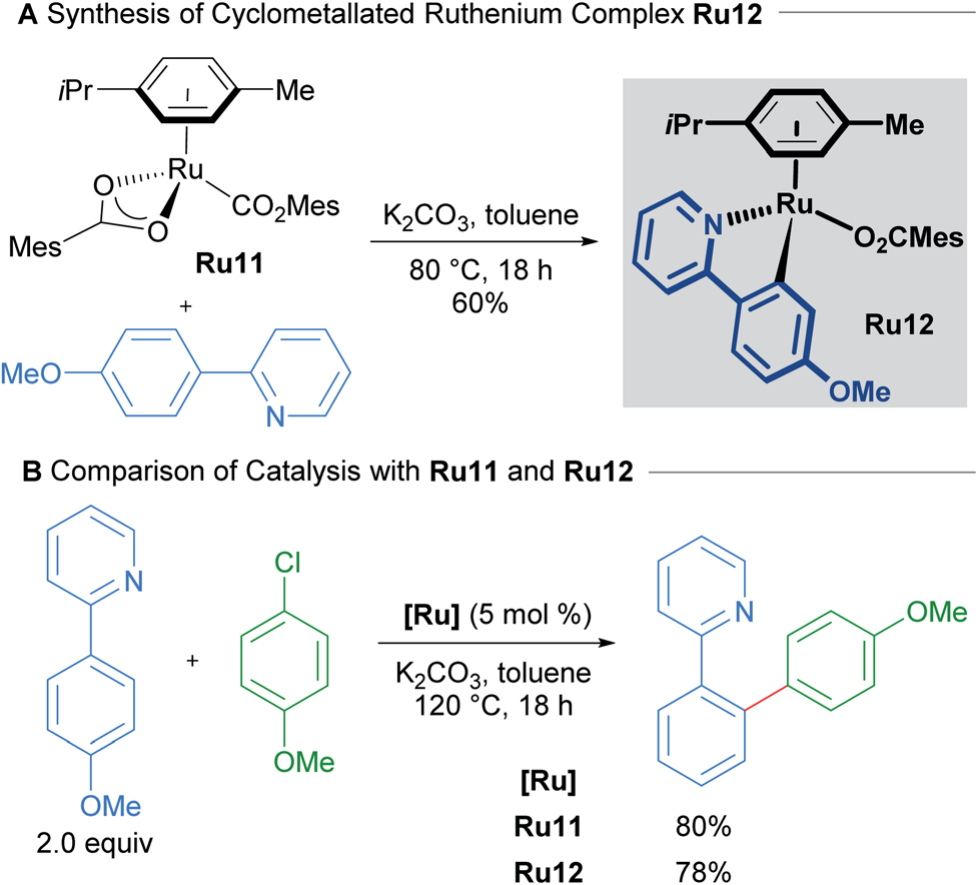
Synthesis of cyclometallated complex Ru12 and use as catalyst.

Work by Ackermann on the direct arylations with ruthenium(ii) carboxylate catalysts has shown that mono-cyclometallated complexes are indeed formed under standard reaction conditions and that these can function as active pre-catalysts (Scheme 6).^25^ A mechanistic study by Dixneuf in 2011 revealed an autocatalytic process in the Ru(ii)-catalysed arylation of arenes, which suggests the facile formation of the cyclometallated species from a Ru(OAc)_2_(*p*-cymene) precatalyst.^26^

**Scheme 7:**
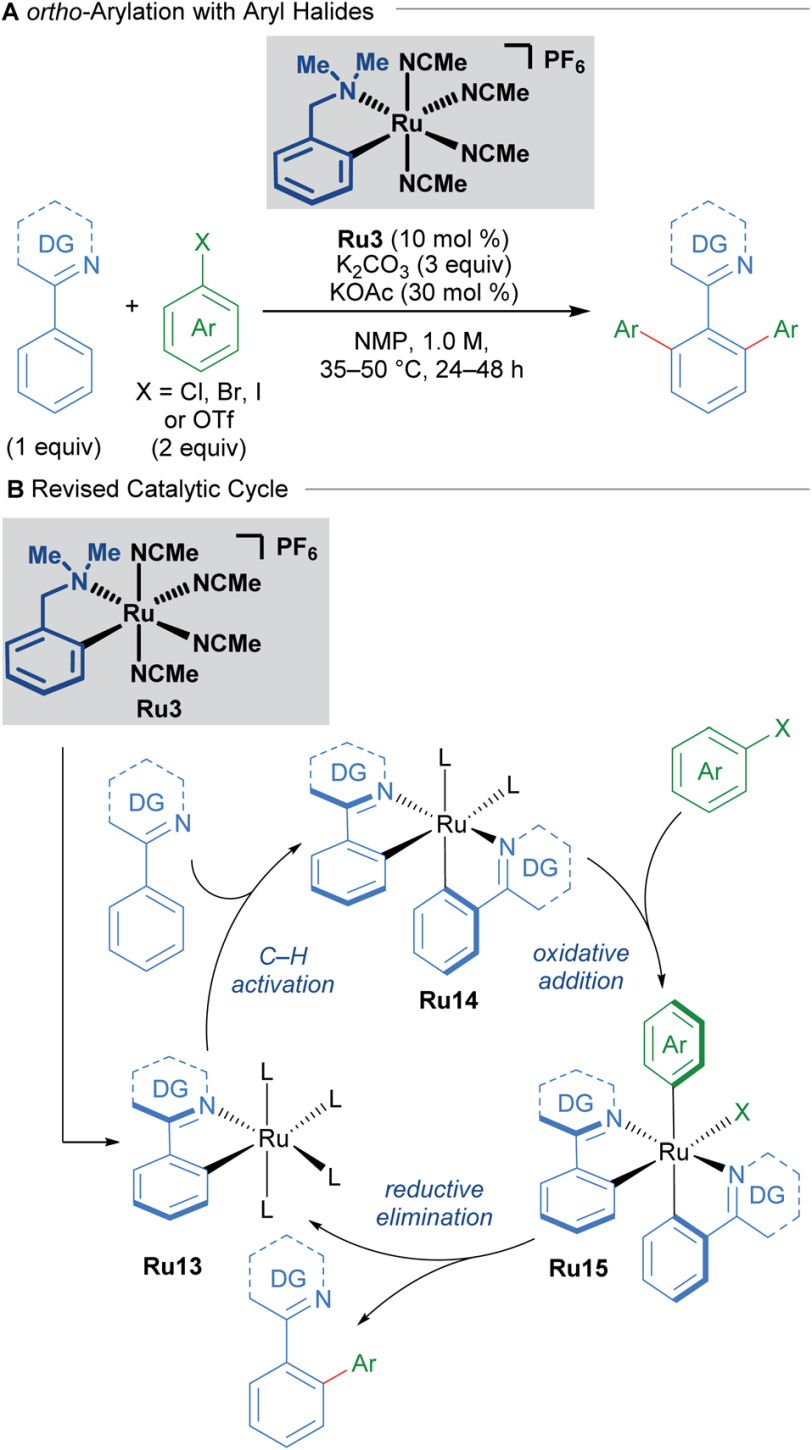
Larrosa's *ortho*-arylation procedure. DG = N-directing group.

A recent study by Larrosa into ruthenium-catalysed late-stage arylations provided further insight into the role of cyclometallated ruthenium complexes (Scheme 7).^27^ The *para*-cymene ligand was found to inhibit the reaction, with high reaction temperatures being necessary solely for promoting its dissociation from the ruthenium centre. After dissociation of the *p*-cymene ligand, the reaction rate accelerates.

These findings led to the development of a new class of mono-cyclometallated precatalysts without η^6^-arene ligands. This new class of catalyst allowed C–H arylation to be run under significantly milder conditions than was previously possible. Consequently, the functional group tolerance was improved, which rendered this method suitable for late-stage arylation of complex molecules. The authors provided evidence for the formation of a bis-cyclometallated ruthenium intermediate (Ru14), which they demonstrated was necessary for oxidative addition of aryl halides. Subsequent reductive elimination from this intermediate affords the coupling product.

Recently, the Ackermann and Greaney groups have demonstrated that these ruthenium-catalysed arylations are also possible at room-temperature with the use of the [RuCl_2_(*p*-cymene)]_2_ precatalyst, under visible-light irradiation conditions.^28,29^ Both groups hypothesise that irradiation of the reaction system with visible light promotes dissociation of the *para*-cymene ligand from the precatalyst, forming the active catalytic species *in situ*. The authors provide evidence for the dissociation of *para*-cymene through the monitoring of free *para*-cymene in the system.

### 2.3 Sulfonation, acylation, and nitration

Ruthenium catalysis offers unique reactivity for the functionalisation of substrates at positions other than *ortho* to the directing group. Such remote functionalisation is enabled by the formation of a ruthenacycle intermediate, which is predisposed towards functionalisation at positions that are distal to the ruthenium–carbon bond. The net result of this form of activation is formal *meta*-functionalisation.

**Scheme 8:**
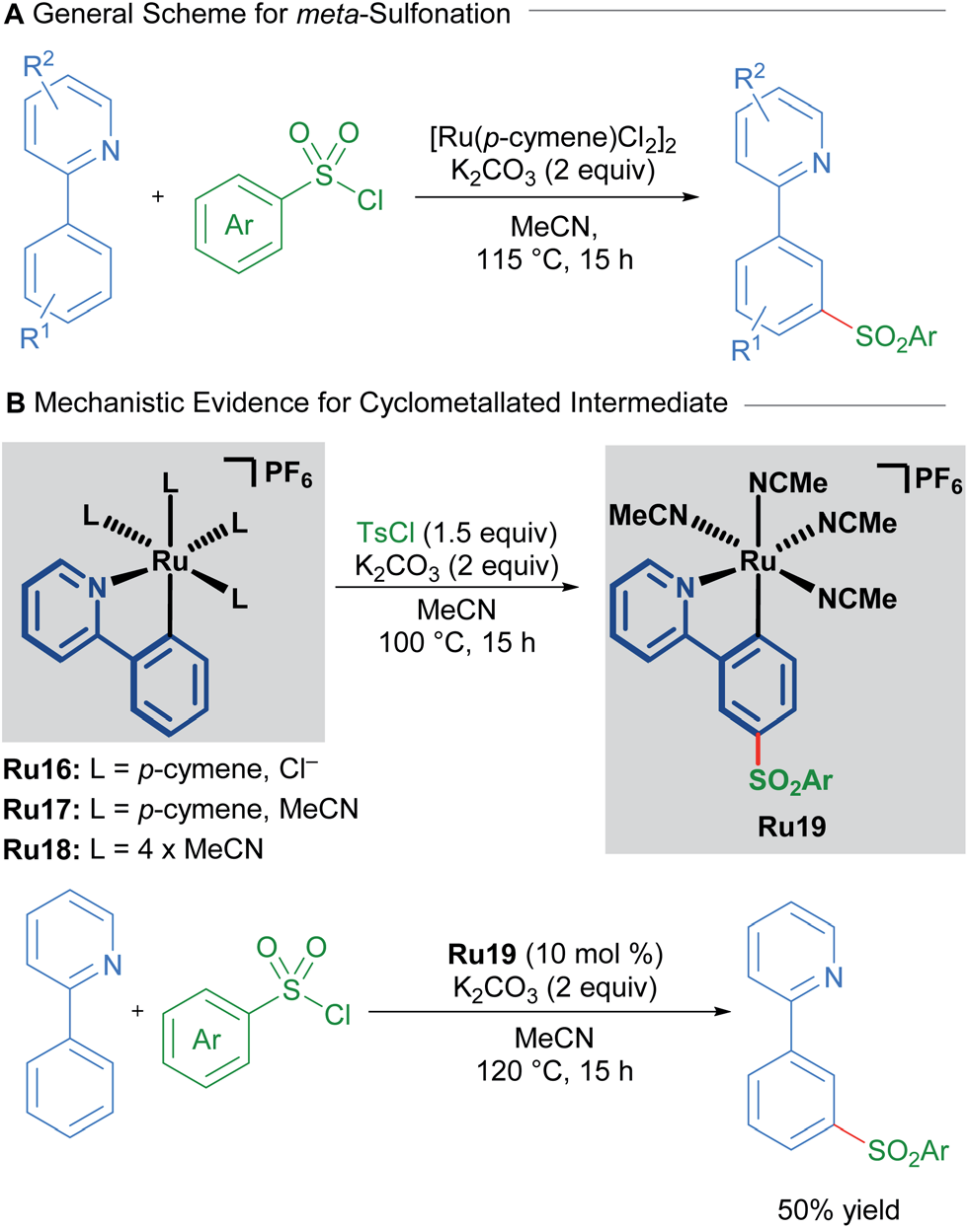
Frost's *meta*-sulfonation procedure.

In a 2011 report, Frost described the first procedure for remote functionalisation of 2-phenylpyridines with ruthenium.^30^ In contrast to previous methods, the ruthenium-catalysed C–H functionalisation reaction between sulfonyl chlorides and 2-phenylpyridines led to *meta*-functionalised products, overriding the traditional *ortho*-selectivity observed with other transition metals. Mechanistic studies indicate that the formation of a ruthenacycle (Ru16, Ru17 or Ru18) is necessary for remote functionalisation, suggesting that ruthenium behaves akin to a classical *ortho*/*para* director in electrophilic aromatic substitution (Scheme 8).^31^

Since this report, various *meta*-functionalisation methods for the installation of alkyl, halide, nitro, and acyl groups have been developed.^32^ Reports by Ackermann into the *meta*-alkylation of arenes containing *N*-heterocyclic directing groups also implicate mono-cyclometallated catalysts. Ackermann shows that these species form under similar conditions to the alkylation reaction, and that these mono-cyclometallated species function as catalysts as well. This work could suggest that these are common intermediates in the catalytic cycle.

Studies into the *meta*-nitration and *meta*-acylation of 2-phenylpyridines by the groups of Zhang and Wang respectively, point towards the involvement of a rare bis-cyclometallated species as an intermediate in the catalytic cycle.^33^

**Scheme 9:**
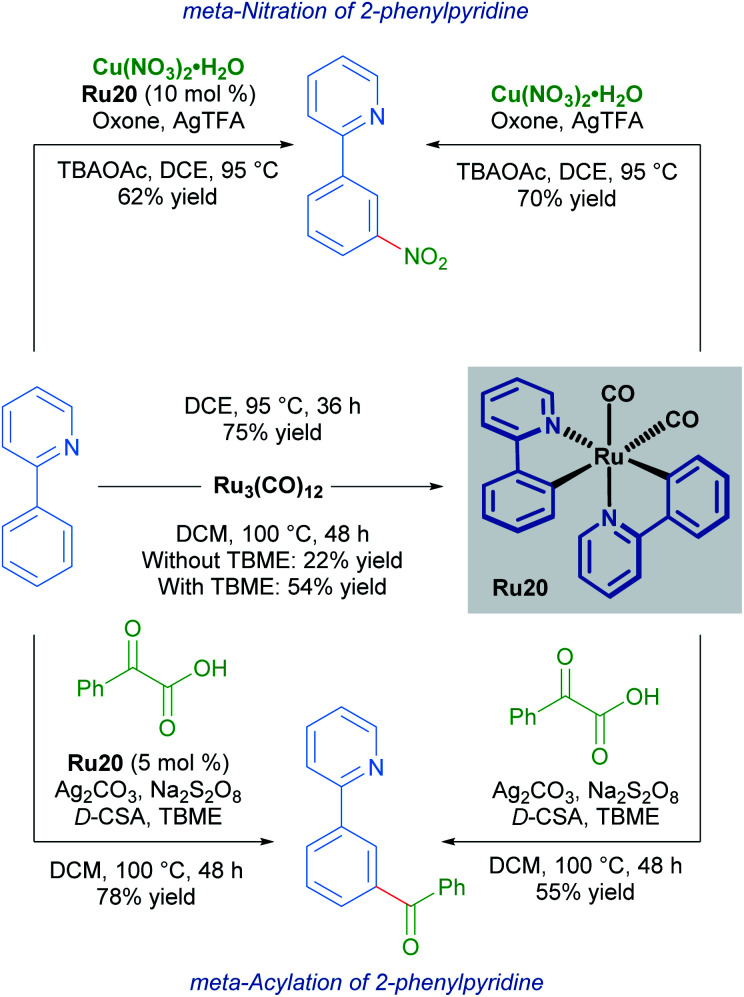
*meta*-Nitration (top) and *meta*-acylation (bottom) *via* bis-cyclometallated species Ru20.

Both groups show that under similar reaction conditions, the same bis-cyclometallated species Ru20 arises from the reaction between 2-phenylpyridine and Ru_3_(CO)_12_. In both cases, the *meta*-functionalised product can be obtained through a stoichiometric reaction from Ru20, or by utilising complex Ru20 in substoichiometric quantities (Scheme 9).

Additional examples of ruthenium-catalysed remote functionalisation are covered in a recent review by Ackermann.^34^

### 2.4 C–H activation/annulation

**Scheme 10:**
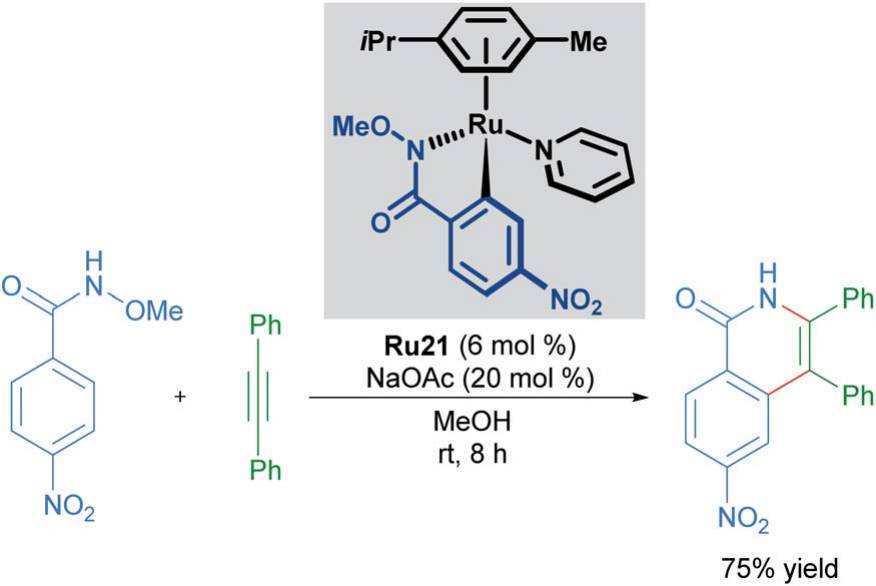
C–H activation and annulation using a cycloruthenated complex.

Cyclometallated ruthenium catalysts have also been demonstrated to be intermediates in C–H activation/annulation reactions involving alkyne insertion. Work by Wang in 2018 demonstrated that cyclometallated complexes are capable of catalysing the formation of an isoquinolinone from the corresponding *N*-methoxy benzylamide and an alkyne (Scheme 10).^35^ These cyclometallated complexes were generated under catalytically similar conditions, and this report suggests that they are involved in the catalytic cycle. Similar work has been carried out by the Jeganmohan group, demonstrating the intermediacy of the mono-cyclometallated intermediates in C–H activation/alkyne annulation reactions.^36^

**Scheme 11:**
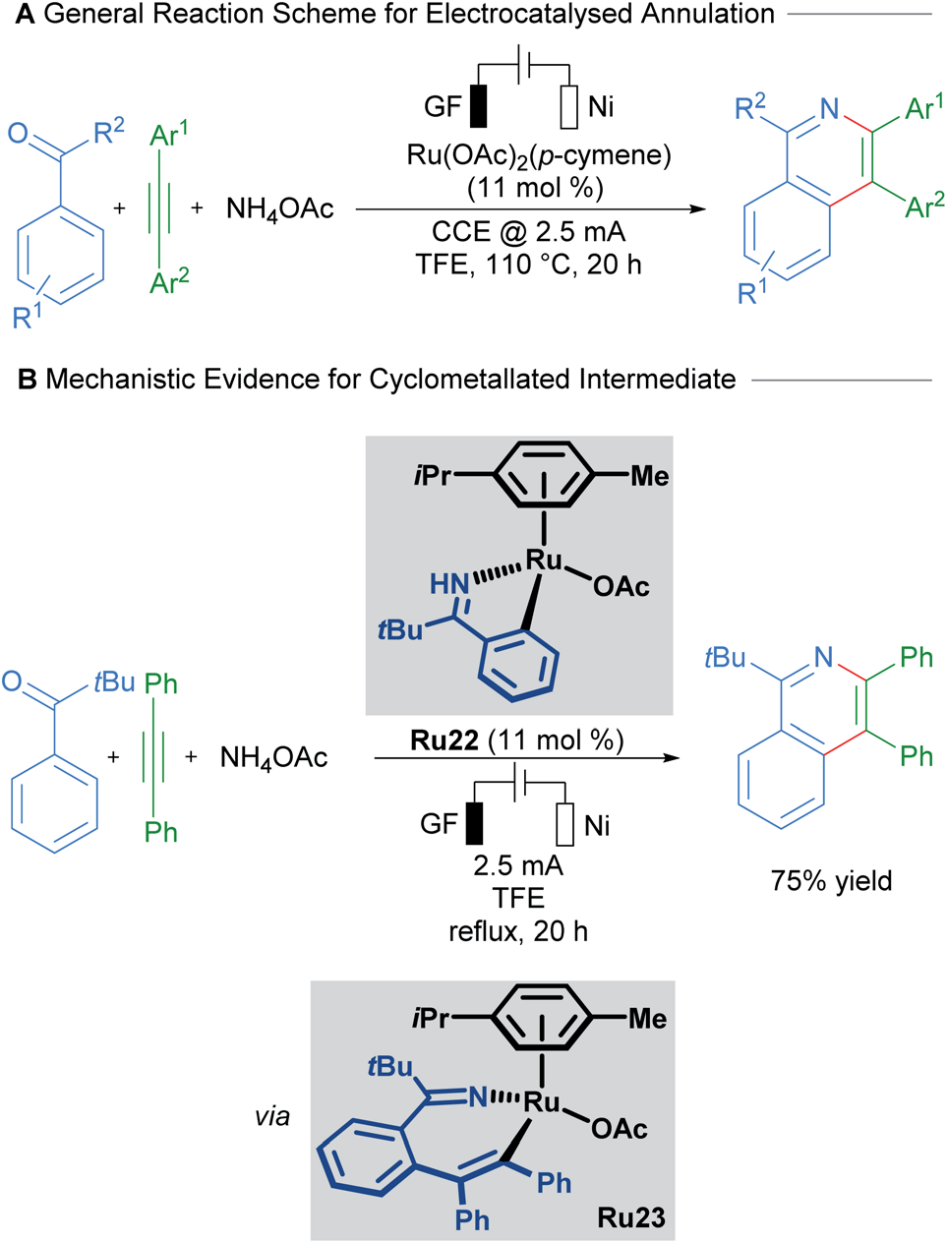
Ackermann's electrocatalysed C–H activation/annulation cascade proceeding through 7-membered ruthenacycle intermediates.

Recent work by Ackermann details the involvement of a cyclometallated ruthenium species in an electrocatalysed C–H activation/ring annulation reaction (Scheme 11).^37^ Here, the cyclometallated species Ru22 was isolated and demonstrated to be an active catalyst in the reaction. Mechanistic studies indicate the formation of 7-membered ruthenacycle Ru23 after addition of an alkyne and before reductive elimination takes place. These results suggest promising applications of ruthenacycles in synthetic electrochemistry.

Mono- and bis- cyclometallated ruthenium species are frequently postulated as catalytic intermediates in various C–H functionalisation processes. While many mono-cyclometallated ruthenium complexes have been reported, the role that bis-cyclometallated ruthenium species play in various catalytic cycles is just beginning to emerge. *In situ* spectroscopic monitoring of stoichiometric reactivity and systematic variation of the cyclometallating moiety with different substituents could provide valuable insight into the behaviour of these species. Future efforts in this area should lead to an improved understanding of how to design effective cycloruthenated complexes for catalytic bond-formation. For a complementary perspective on the involvement of metallacycles in C–H activation, readers are directed to a recent review by Wencel-Delord and coworkers.^38^

## 3. Chiral-at-ruthenium catalysis

Catalytic asymmetric synthesis of organic molecules relies on metal catalysts with chiral ligands coordinated to the metal centre. These transformations are desirable because the metal and the chiral ligands are used in catalytic quantities compared to the use of chiral auxiliaries, which are required in stoichiometric quantities. To date, a vast array of chiral metal complexes has been developed for a wide variety of catalytic asymmetric transformations. Within the area of asymmetric catalysis, another strategy that is underexplored is the use of octahedral chiral-at-metal complexes of cobalt, iridium, rhodium and ruthenium.^39^ These complexes are optically active, despite their bidentate achiral ligands, due to metal-centred, octahedral centrochirality. Additionally, these complexes typically possess labile ligands such as acetonitrile, which displace readily. This permits facile ligand exchange with the substrate molecule, thereby facilitating catalytic processes. In this section, we will cover the pioneering and recent advancements made by the Meggers group in developing the chemistry of chiral-at-ruthenium complexes. Asymmetric metathesis reactions catalysed by chiral-at-ruthenium complexes published by Grubbs will be discussed in section 4.

**Scheme 12:**
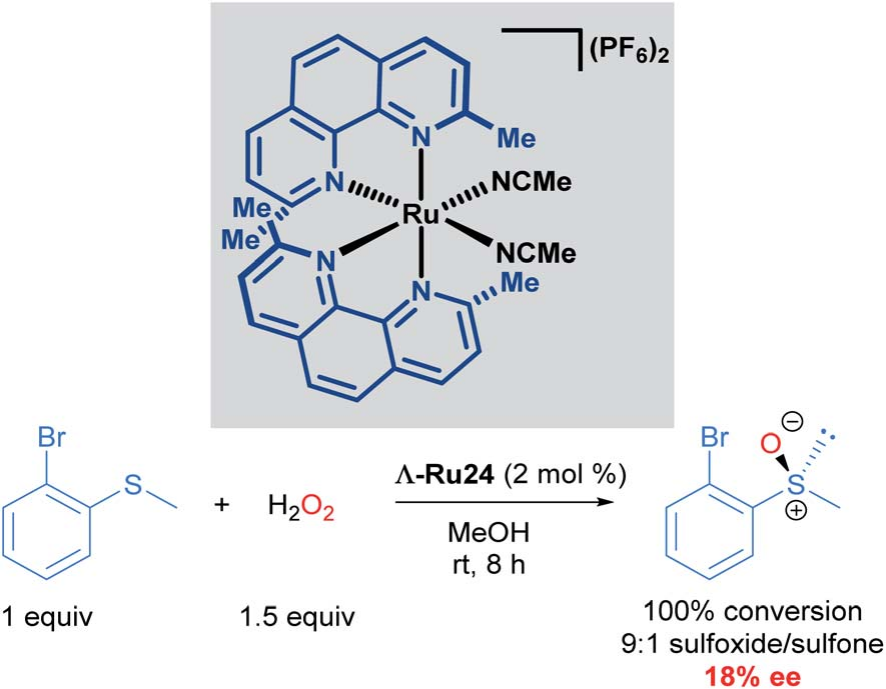
First use of a chiral-at-ruthenium complex in asymmetric catalysis.

In 2003, Fontecave described the first chiral-at-ruthenium complex used in asymmetric catalysis (Λ-Ru24).^40^ In this work, the oxidation of 2-bromophenyl methyl sulphide to the corresponding enantioenriched sulfoxide was reported with a low, but promising 18% ee, serving as important proof-of-concept for using octahedral metal-cantered chirality for asymmetric induction (Scheme 12).

**Scheme 13:**
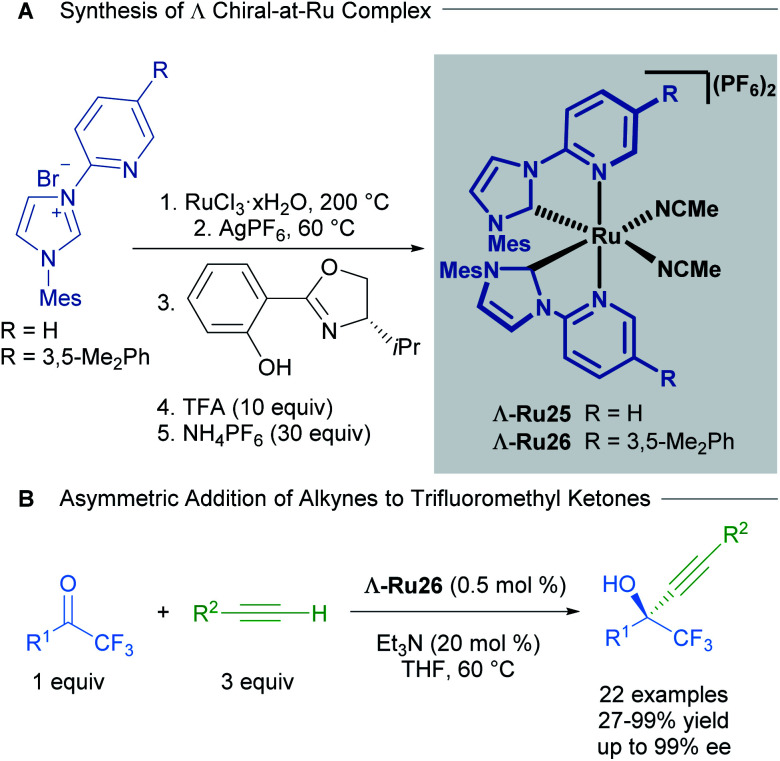
Enantioselective alkynylation of trifluoromethyl ketones catalysed by chiral-at-ruthenium complexes.

Based on extensive work with chiral-at-rhodium and iridium complexes, Meggers and co-workers have also published the vast majority of recent examples with chiral-at-ruthenium complexes.^41^ In a report from 2017, Meggers described the synthesis, characterisation, stability studies and catalytic activity of a cationic, bisacetonitrile ruthenium complex with two *N*-(2-pyridyl)NHC ligands.^42^ The synthesis of the complex began with the reaction of RuCl_3_ hydrate with an *N*-(2-pyridyl)imidazolium salt in ethylene glycol at 200 °C, followed by salt metathesis with AgPF_6_ at 60 °C to obtain the racemic complex. The racemic mixture was resolved using a chiral auxiliary, affording diastereomerically pure complexes Λ and Δ. Treatment with TFA afforded the enantiomerically pure complexes Λ-Ru25 and Λ-Ru26 (Scheme 13A). The absolute configuration of the complexes was assigned by a combination of circular dichroism and an X-ray crystal structure of a derivative of Δ-Ru26. No isomerisation or decomposition on heating these complexes in THF over 72 h was observed, suggesting exceptional stability.

The enantioselective alkynylation of trifluoromethyl ketones was carried out to test the catalytic activity of the complexes Λ-Ru26 and Δ-Ru26 (Scheme 13B). Both enantiomers outperformed previously reported chiral-at-metal rhodium and iridium complexes, yielding high reactivity and enantioselectivity in this transformation. The reaction tolerated a wide variety of substituents in both the alkyne and the ketone, with high yields and enantioselectivity (both >91%); however, phenethyl and ester groups in the ketone afforded 62% and 7% ee respectively. In addition, the authors were able to synthesise a chiral intermediate for the synthesis of the AIDS treatment drug Efavirenz in 58% yield and 92% ee. A year later, Houk, Meggers and co-workers published a computational study on this reaction.^43^ The proposed mechanism invokes pre-coordination of both the ketone and alkyne to the metal centre, followed by the formation of a ruthenium acetylide and its subsequent addition to the coordinated ketone. This pre-coordination is thought to be key for high levels of asymmetric induction. The calculated free energies agreed with the experimentally observed enantioselectivities.

**Scheme 14:**
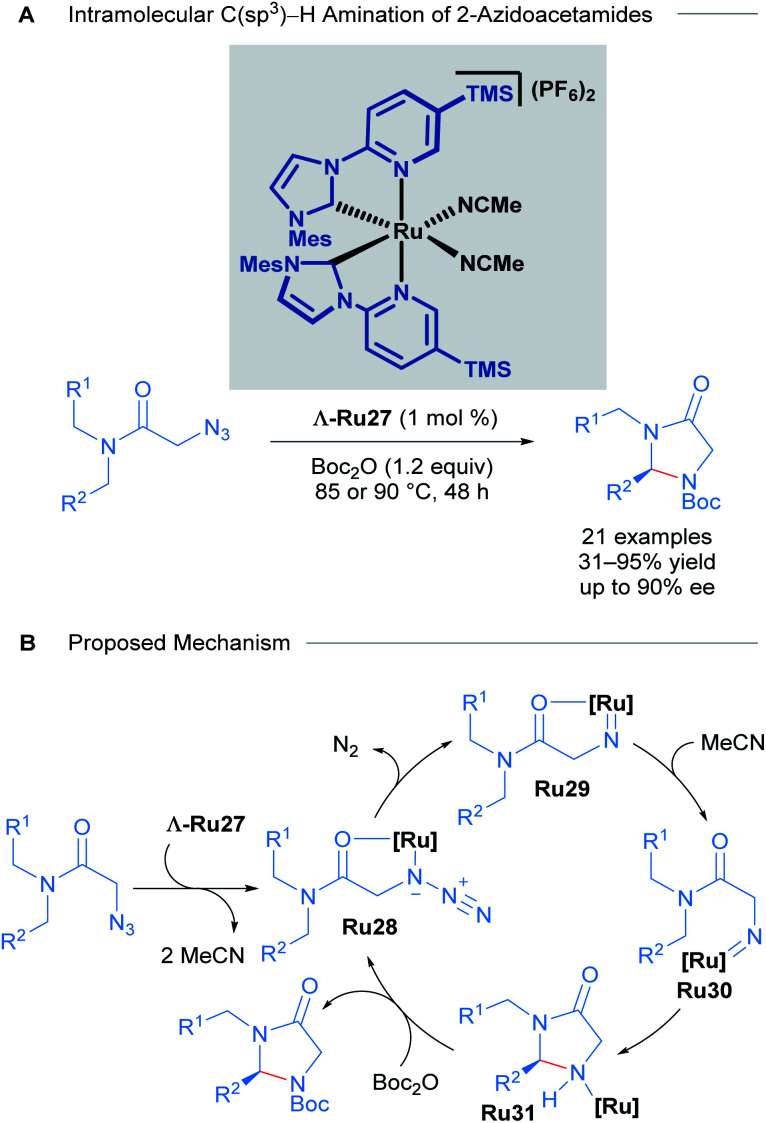
Ru catalysed intramolecular C(sp^3^)–H amination of azidoacetamides (A) and proposed mechanism of the reaction (B).

With these impressive results in asymmetric induction using the Λ-Ru26 catalyst, Meggers later developed a catalytic enantioselective intramolecular C(sp^3^)–H amination of 2-azidoacetamides using slightly modified complexes. The 3,5-Me_2_Ph substituent in the pyridyl R group of the NHC ligand was exchanged for a TMS group to yield highly reactive catalysts (Scheme 14).^44^ 15 chiral imidazolidin-4-ones and 6 tricyclic compounds were obtained with good yields (31 to 95%) and ee (close to 90%) in 18 of the 21 reported examples (Scheme 14A). A thorough mechanistic study was carried out and supported by DFT calculations (Scheme 14B). The proposed mechanism begins with ligand exchange of two acetonitrile molecules, leading to bidentate coordination of the starting material to the ruthenium catalyst. Subsequent extrusion of N_2_ from organic azide group generates a ruthenium-imido intermediate that de-coordinates the amide group and performs a stereo-controlled insertion into a C–H bond, forming the catalyst-bound imidazolidine-4-one. Finally, this intermediate reacts with Boc_2_O to turn over the catalyst and release the final product.

**Scheme 15:**
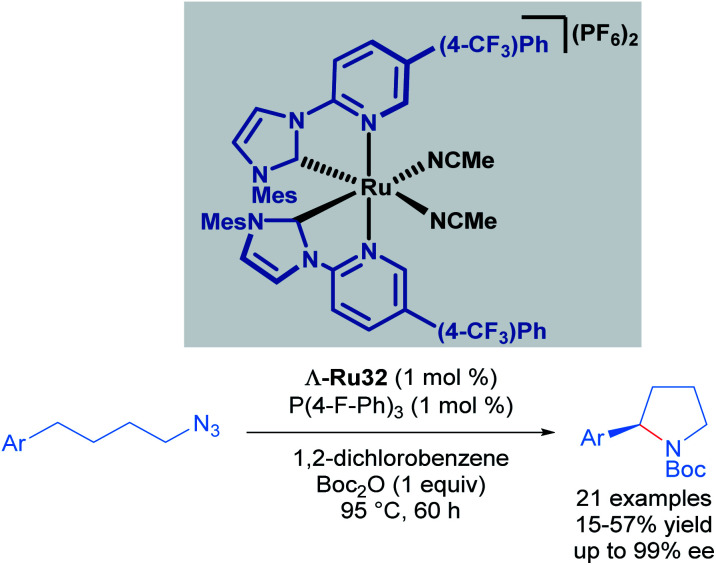
Intramolecular C(sp^3^)–H amination of alkylazides.

Recognising the potential of this C–H transformation, the authors used a similar approach in the formation of chiral pyrrolidines with a dual catalytic system composed of a chiral-at-ruthenium catalyst in combination with triarylphosphines (Scheme 15).^45^ The phosphine was used in the activation of the alkyl azide. Although the synthesis of 2-aryl pyrrolidines from aliphatic azides has been reported before, this was the first example proceeding with high enantioselectivity (up to 99% ee), albeit in moderate yields (15–57%). These low yields are due to the formation of linear Boc-protected amines as side products and incomplete conversions for the reported examples. The authors also performed mechanistic experiments. Reacting the starting material and the phosphine, (2 hours at 95 °C) afforded a catalytically competent iminophosphorane *via* the Staudinger reaction. In addition, when the reaction was carried out without added phosphine, the yield decreased drastically even with prolonged reaction times. The proposed mechanism is similar to the one shown previously (Scheme 14), with the inclusion of a pre-activation step (conversion of the azide to the iminophosphorane *via* the Staudinger reaction). Thus, the ruthenium-imido intermediate is generated *via* nitrene transfer from the iminophosphorane to the ruthenium complex.

**Scheme 16:**
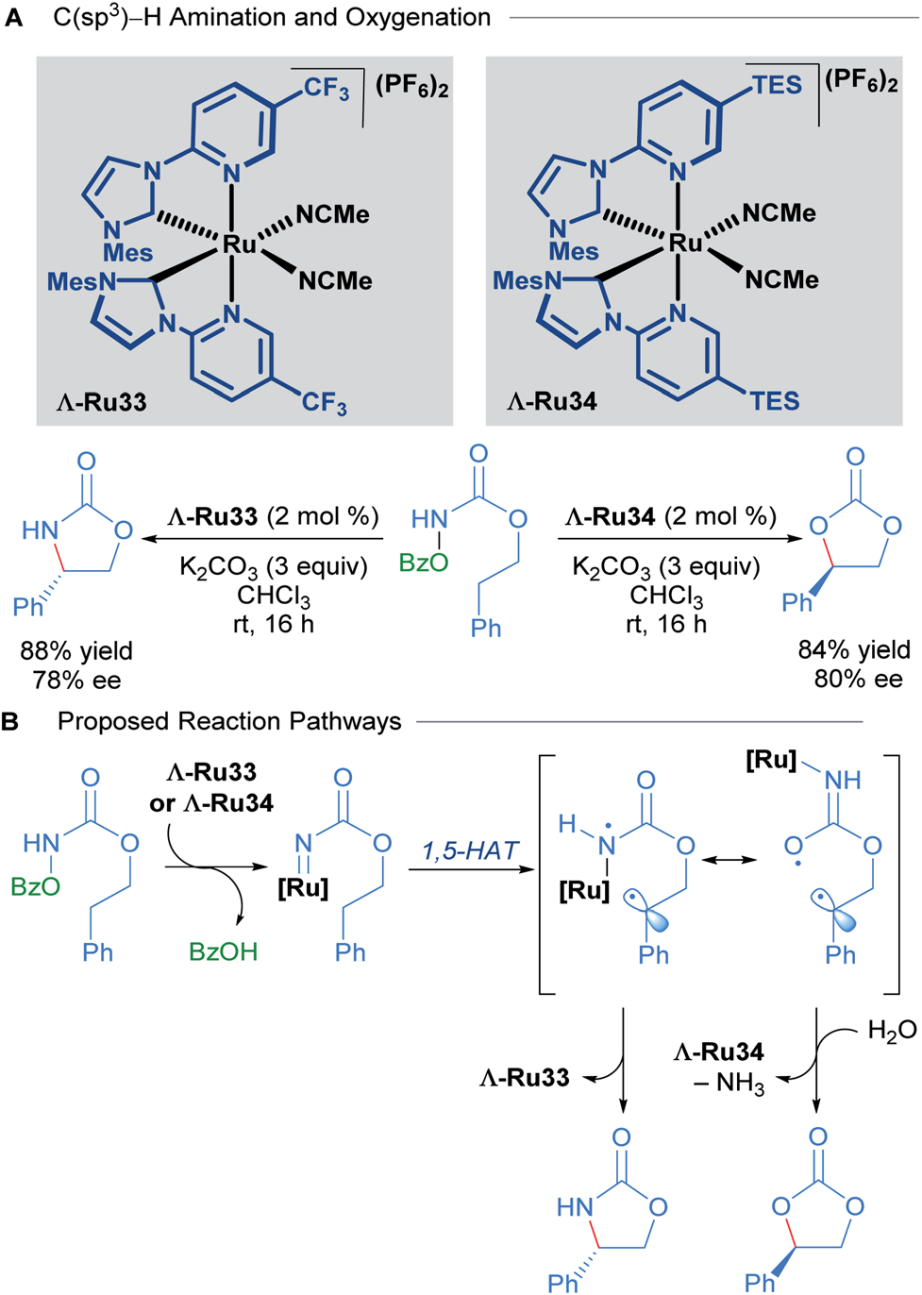
Asymmetric intramolecular C(sp^3^)–H amination and oxygenation (A) and proposed reaction pathways (B).

Recently, Meggers and co-workers extended the use of this type of *N*-(2-pyridyl)NHC-derived ruthenium catalysts to C(sp^3^)–H activation of carbamates (Scheme 16).^46^ Through a simple change in the substituents of the catalyst, they were able to attain C–N and C–O bond formation *via* nitrene and carbene insertions respectively. Since there was extensive literature precedent for ruthenium-catalysed nitrene insertions of carbamates, the authors focused instead on developing the C(sp^3^)–H oxygenation reaction to obtain cyclic carbonates. The scope of this reaction was performed with a racemic mixture of the optimal chiral-at-ruthenium complex (Ru34), yielding the desired carbonate products in good to excellent yields. In addition, hydrolysis of the carbamates with NaOH yielded 1,2-diols in good yields.

To explore the enantioselective variant of this reaction, the enantiomerically pure complexes were synthesised with the aid of chiral auxiliaries. The Λ isomers, Λ-Ru33 and Λ-Ru34, were applied to C–O and C–N bond formation, affording the corresponding carbonate (84% yield, 80% ee) and carbamate (88% yield, 78% ee) respectively (Scheme 16A). In the mechanistic proposal for this process, the reaction begins with the formation of a ruthenium nitrenoid, followed by 1,5-hydrogen atom transfer to generate a diradical intermediate (Scheme 16B). The diradical intermediate can react directly with the nitrogen to form a cyclic carbamate. Alternatively, if the diradical is sufficiently long-lived, a conformational change can occur, shifting the ruthenium catalyst away from the benzylic radical, leading to radical–radical recombination at the oxygen atom instead. The preferential formation of C–O to C–N bonds in this instance was attributed to the steric bulk conferred by the TES groups of Λ-Ru34, suppressing the previously established C–N bond formation. Subsequent hydrolysis of the exocyclic imine (not shown) affords the cyclic carbonate product.

**Scheme 17:**
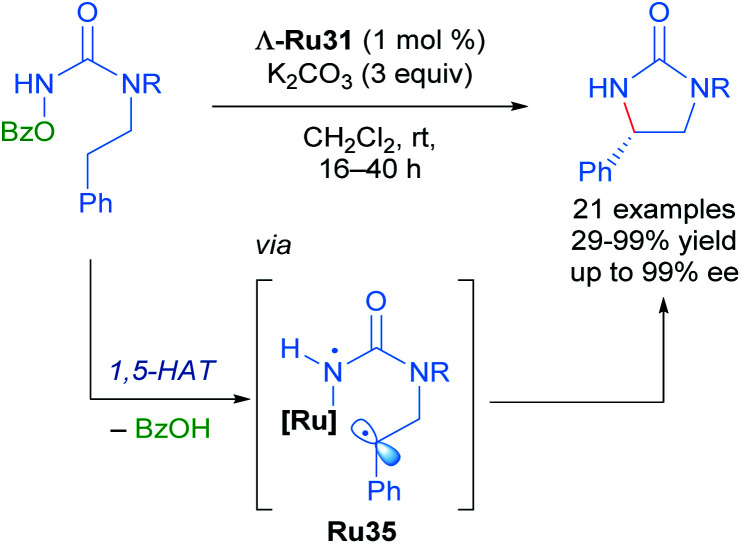
C–H amination of urea derivatives.

With these impressive results in hand, Meggers extended this same approach to the enantioselective ring-closing C–H amination of urea derivatives (Scheme 17).^47^ The authors obtained diverse cyclic ureas with excellent yields in nearly all cases and with enantioselectivity up to 99%. In addition, they were also able to use this method to obtain different chiral intermediates in the synthesis of medicinal agents, natural products and chiral catalysts.

**Scheme 18:**
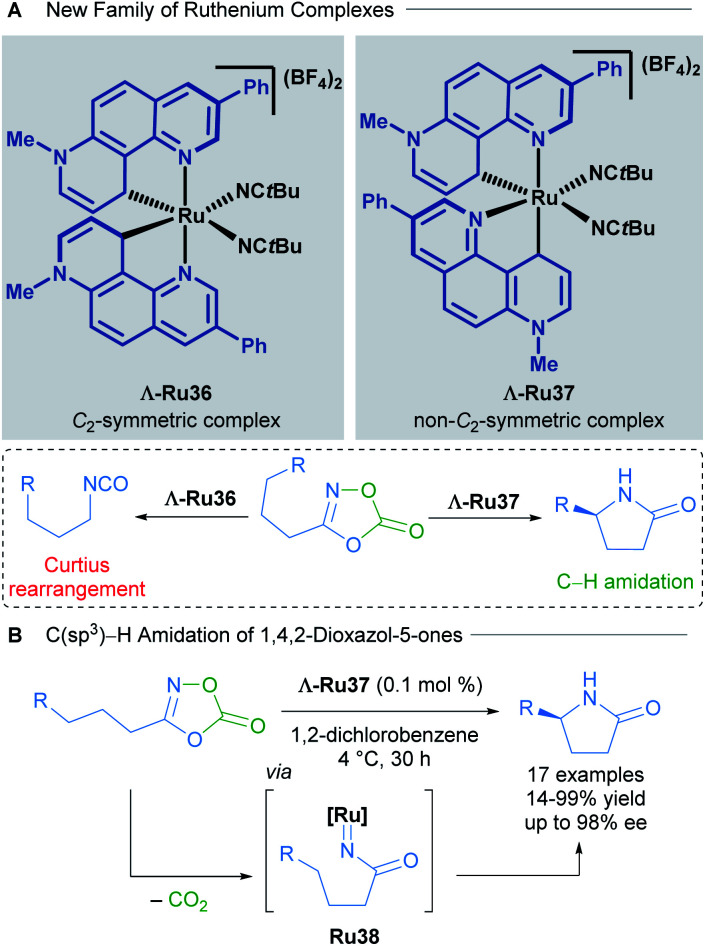
C_2_-symmetric and non-C_2_-symmetric new complexes (A) and C(sp^3^)–H amidation of 1,4,2-dioxazol-5-ones (B).

The development of new chiral-at-ruthenium catalysts was also achieved by changing the nature of the NHC ligand. In 2019, Meggers and co-workers described a new family of chiral-at-ruthenium complexes with two phenanthrolinium-type ligands.^48^ These new catalysts were applied in the enantioselective intramolecular C(sp^3^)–H amidation of 1,4,2-dioxazol-5-ones to form chiral lactams (Scheme 18). The authors observed that when both NHC ligands were coordinated to the metal centre forming a C_2_-symmetric complex (Λ-Ru36), the major product was the result of an undesired Curtius rearrangement of the starting material (Scheme 18A). On the other hand, the non-C_2_-symmetric diastereomeric complex Λ-Ru37 catalysed the desired reaction exceptionally well, delivering enantioselectivities up to 98% ee (Scheme 18B). In the optimisation of this reaction, the authors compared the activity of these new complexes with the previously described Λ-Ru22, Λ-Ru24 and Λ-Ru33, obtaining in these three cases only the undesired Curtius rearrangement product. With 0.1 mol% of catalyst Λ-Ru37, 17 chiral lactams were obtained (14–99% yield, 71 : 29 to 99 : 1 er). A gram scale reaction was also demonstrated employing a low catalyst loading of 0.005 mol%, affording the final product in 56% yield, with an impressive and unprecedented TON of 11 200. DFT calculations revealed that both the strong electron-donating character of the phenanthrolinium ligand in addition to its non-C_2_-symmetric arrangement in the coordination sphere are crucial for providing an electron-rich, nucleophilic ruthenium nitrene intermediate that evolves *via* a C–H activation pathway. The absolute configuration of the final product was dependent upon octahedral metal-centred chirality (Λ or Δ) as detailed in previous reports.

**Scheme 19:**
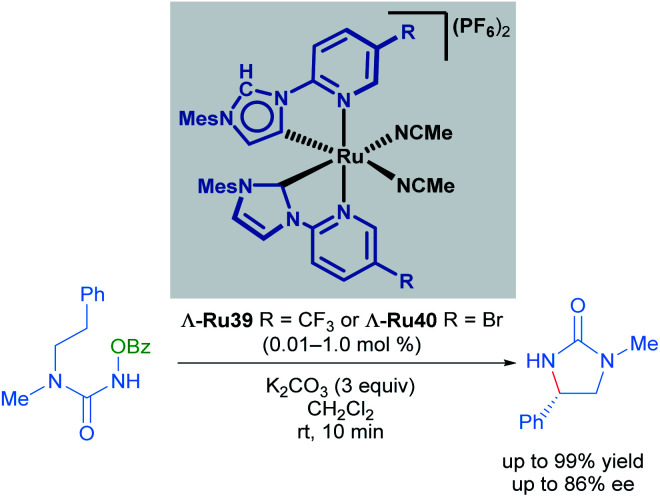
C–H amination of urea derivative catalysed by normal and abnormal NHC coordination complexes.

Very recently, Meggers also described the synthesis of mixed normal/abnormal NHC complexes of chiral-at-ruthenium catalysts (Scheme 19).^49^ In this report, the previously employed *N*-(2-pyridyl)NHC ligand was coordinated to the metal centre in two different ways: one of the ligands is linked to the metal centre by the C2 carbon of the imidazole heterocycle (normal coordination mode) while the other ligand was linked to the C4 carbon in an abnormal coordination mode. After optimisation of the catalyst synthesis, the new family of abnormally bound NHC complexes was found to display higher reactivity in the enantioselective ring-closing C–H amination of urea derivatives than the C_2_-symmetric complexes used previously.^47^ Unfortunately, lower enantioselectivity was obtained with these complexes.

In conclusion, the continued study of chiral-at-ruthenium complexes offers new strategies towards the synthesis of chiral molecules. The outstanding work of the Meggers research group over the last 5 years in this area demonstrates the impact these types of cycloruthenated complexes are beginning to have on asymmetric catalysis, inviting future improvements in catalyst design as well as new asymmetric reactions with this class of catalyst.

## 4. *Z*-selective olefin metathesis

Since the first examples of ruthenium-catalysed olefin metathesis appeared in 1965,^50,51^ many significant advancements in terms of catalyst design and substrate scope have been realised and described in the literature. The 2005 Nobel Prize in Chemistry was awarded to Robert H. Grubbs, Richard R. Schrock, and Yves Chauvin, in recognition of the importance of olefin metathesis in modern organic synthesis. Several reviews in this field have been published, summarising the last 20 years of ruthenium-catalysed metathesis reactions such as: cross metathesis (CM) ring-opening/cross metathesis (ROCM), ring-closing metathesis (RCM) and ring-opening metathesis polymerisation (ROMP).^8*c*,52^ The vast majority of the published examples describe the use of Hoveyda–Grubbs first and second generation catalysts. Since this review aims to showcase the use of cycloruthenated complexes as catalysts in various transformations, we have focused our attention on cyclometallated adamantyl-NHC ruthenium complexes in *Z*-selective metathesis reactions.

**Scheme 20:**
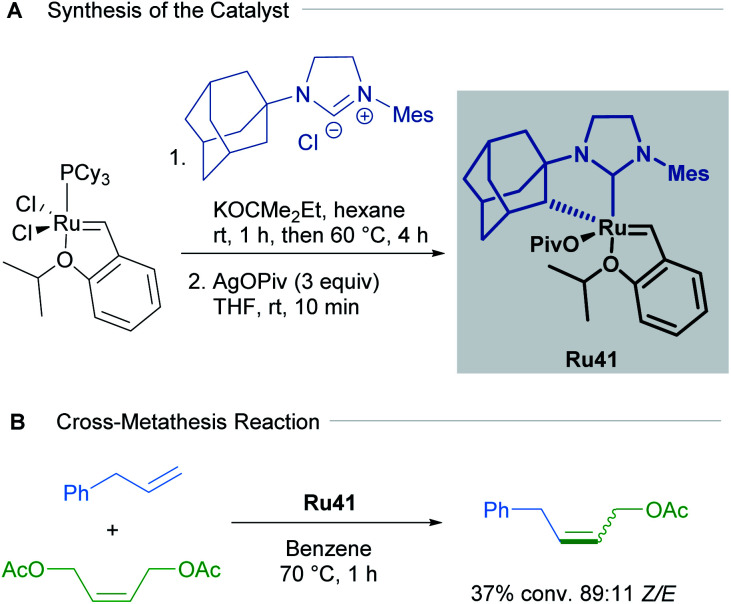
First cycloruthenated complex in *Z*-selective olefin metathesis.

The first cycloruthenated catalyst for *Z*-selective olefin metathesis reactions was described by Grubbs in 2011.^53^ The complex possesses an adamantyl-derived *N*-heterocyclic carbene (NHC) that chelates the metal centre *via* cyclometallation (Scheme 20A). The catalyst Ru41 was examined in the CM reaction of allylbenzene with *cis*-1,4-diacetoxy-2-butene to test its efficiency relative to a related ruthenium complex, which is cyclometallated to a mesityl ring (Scheme 20B). The *Z*-selectivity obtained with Ru41 was 10 times greater than with its mesityl analogue. Additionally, over the course of reaction optimisation, the authors observed that catalyst Ru41 tolerated the presence of water in the reaction media. Anhydrous conditions for sensitive polymerisations were potentially unnecessary. Nevertheless, degassing of the solvent was mandatory since oxygen was detrimental to the reaction.

**Scheme 21:**
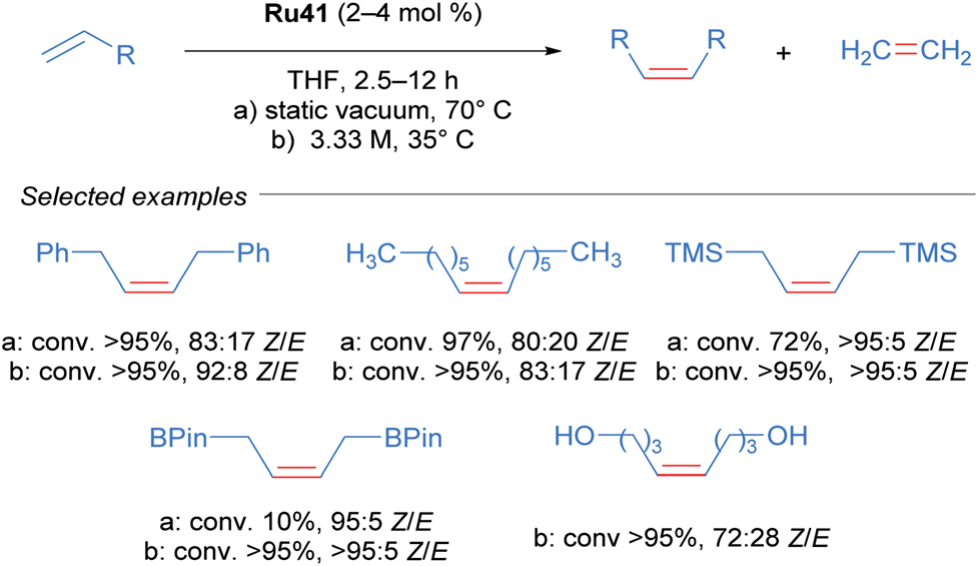
*Z*-selective olefin metathesis of different alkenes using Ru41 as catalyst.

Realising that complex Ru41 could catalyse the CM reaction with high *Z*-selectivity, the same research group applied this catalyst in the homodimerisation reaction of different olefins (Scheme 21).^54^ The complex was effective at 70 °C in THF and acetonitrile under static vacuum, giving high conversion and selectivity. The ethylene generated as the reaction progressed was removed by this vacuum, since an atmosphere of ethylene lead to catalyst decomposition. Alternatively, when the reaction was performed at 35 °C in THF, the concentration of olefin was increased to render the removal of ethylene unnecessary. Under these conditions, an improvement in both reactivity and selectivity of the reaction was observed. The homodimerisation of allyl pinacol borane was also achieved, a substrate that otherwise displayed little reactivity at 70 °C. More challenging substrates such as alcohols and amines were also successful. Lastly, the authors highlighted the stability and reactivity of Ru41 at room temperature in various solvents.

**Scheme 22:**
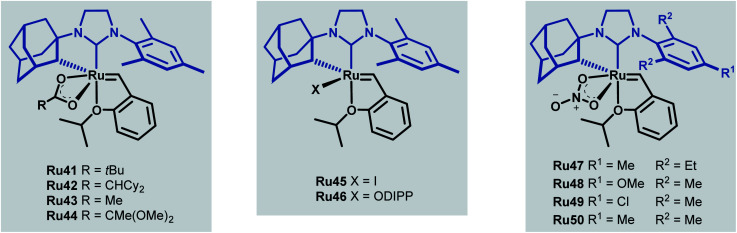
New cycloruthenated NHC complexes for cross metathesis reactions.

Towards improving the effectiveness of cyclometallated adamantyl-NHC complexes in *Z*-selective olefin metathesis, Grubbs and co-workers decided to alter the carboxylate ligand and the aryl group in the NHC moiety of catalyst Ru41.^55^ They were able to obtain ten different complexes, each of which were evaluated for improvements in performance across different CM reactions (Scheme 22).

**Scheme 23:**
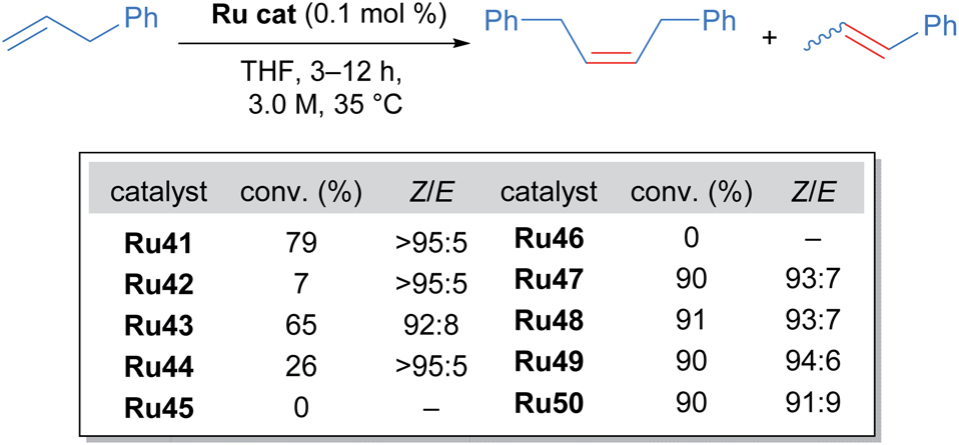
Homodimerisation of allylbenzene with new cycloruthenated catalysts.

The *tert*-butyl carboxylate ligand was systematically replaced with carboxylates bearing different alkyl groups (Ru42, Ru43 and Ru44). Substitution with other types of ligands, such as: iodide (Ru45), 2,6-diisopropylphenoxide (ODIPP, Ru46), and nitrato ligands (coordinating in κ^2^ fashion) was also explored. Minor variations in the aromatic ring of the NHC were examined as well (Ru47, Ru48, Ru49 and Ru50). To clearly evaluate the effect of these changes on catalyst performance in CM reactions, a 0.1 mol% catalyst loading was employed. Furthermore, a high concentration of olefin substrate was required to avoid the aforementioned catalyst deactivation by ethylene. Initially, the homodimerisation reaction of allyl benzene was studied with each of the synthesised catalysts (Scheme 23).

**Scheme 24:**
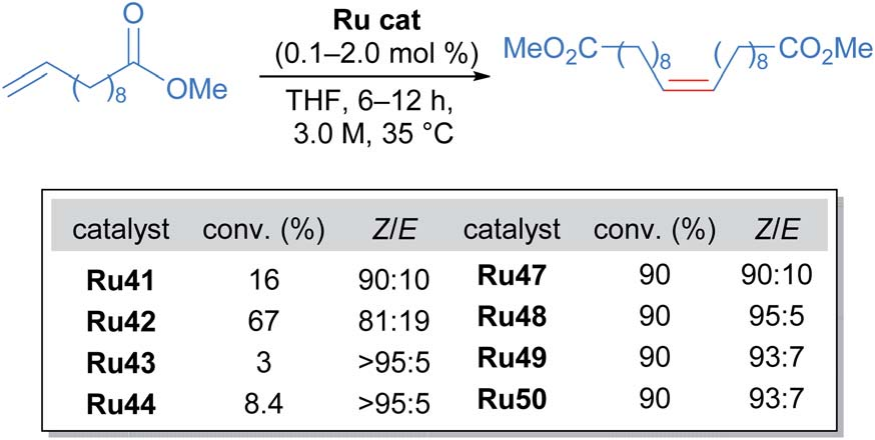
Homodimerisation of challenging olefins with new ruthenium catalysts.

In this case, the desired cross metathesis product was detected alongside the side product of olefin isomerisation. This undesired isomer was observed in all reactions, and it was the only observable product in the case of catalysts Ru45 and Ru46 (with iodide and phenoxide ligands respectively). In the remaining cases, a variable amount of side product was observed, and the *Z*-selectivity was greater than 81%. With these promising results, the authors evaluated the more challenging alkene 10-undecenoate in the CM dimerisation reaction, employing the same catalysts that were effective in the reaction of allylbenzene (Scheme 24).

**Scheme 25:**
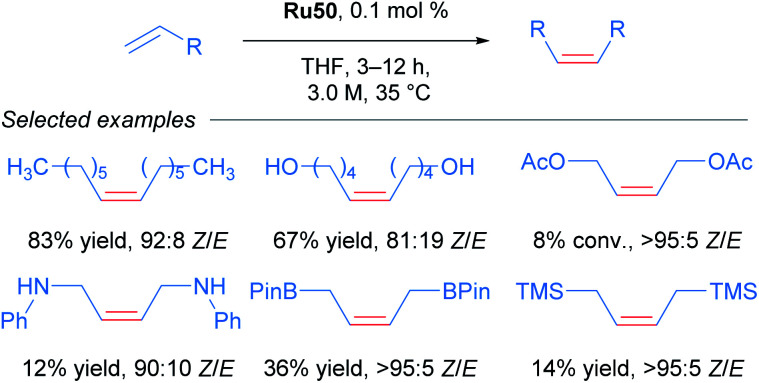
Alkene scope with nitrato-based catalyst Ru50.

The carboxylate-derived catalysts Ru41, Ru42, Ru43 and Ru44 were found to possess low reactivity but high *Z*-selectivity, with Ru42 being active only when 20 times the normal catalyst loading was used. On the other hand, the nitrato complexes Ru47 to Ru50 displayed exceptional conversions (>90%) and excellent *Z*-selectivities (up to 95 : 5 *Z*/*E*). In addition, to demonstrate the superior performance of nitrato complexes, several homodimerisation reactions were carried out (Scheme 25).

**Scheme 26:**
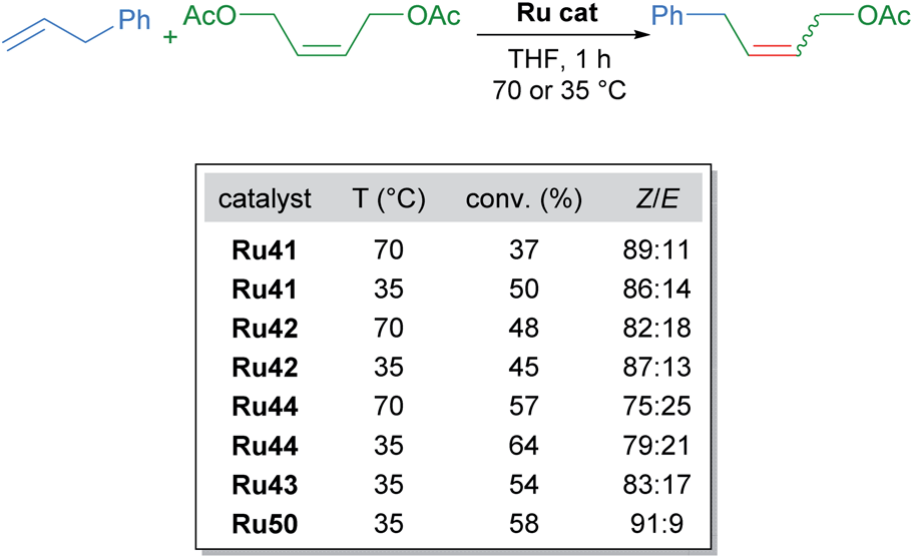
Cross metathesis with allylbenzene and bis-acetate olefin.

Lastly, the cross-metathesis of allylbenzene with *cis*-1,4-diacetoxy-2-butene was also successful (Scheme 26). The new family of nitrato-based complexes displayed higher reactivity and *Z*-selectivity than Ru41 for unsymmetrically substituted alkenes, laying the foundations for future work in this field.

**Scheme 27:**
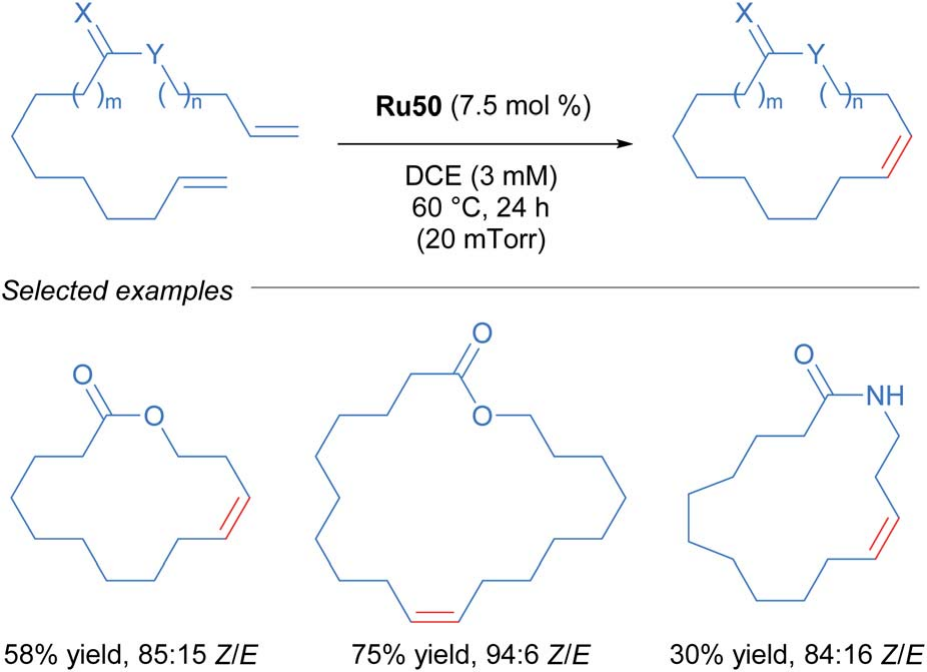
*Z*-selective metathesis for macrocyclisation.

After the publication of these ruthenium complexes as highly efficient catalysts for *Z*-selective metathesis reactions, nitrato ruthenium complexes have been used in several metathesis reactions with a wide variety of olefins. In 2013, Grubbs and co-workers described the first *Z*-selective macrocyclisation using Ru50 (Scheme 27).^56^ In this report, the authors were able to obtain the macrocycle *via* ring-closing metathesis. In addition, using the same catalyst Ru50 but performing the *Z*-ethenolysis of a mixture of *Z* and *E* macrocycles, they obtained the *E* isomer exclusively. In the case of the *Z*-selective metathesis, the desired macrocycles were obtained in moderate to good yields (30–75%) and with good to excellent *Z*-selectivity (68–94%) tolerating various functional groups such as alcohols, acetals and amides.

**Scheme 28:**
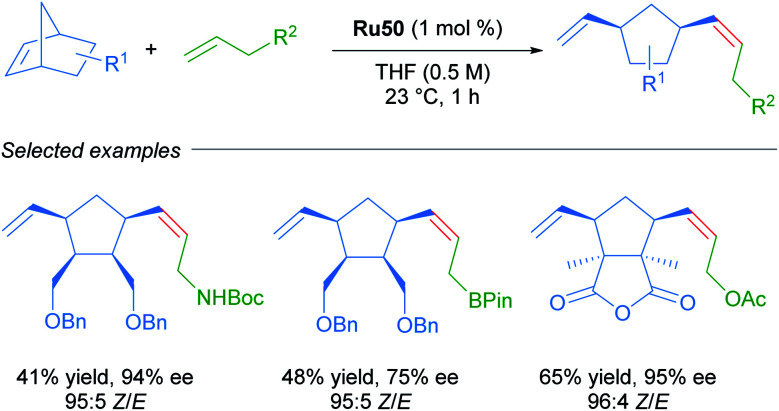
Asymmetric ring-opening/cross metathesis.

Due to the importance of chiral molecules in organic and medicinal chemistry, they also described the asymmetric ring opening-cross metathesis (AROCM) using chiral-at-metal Ru50 catalyst (Scheme 28).^57^

**Scheme 29:**
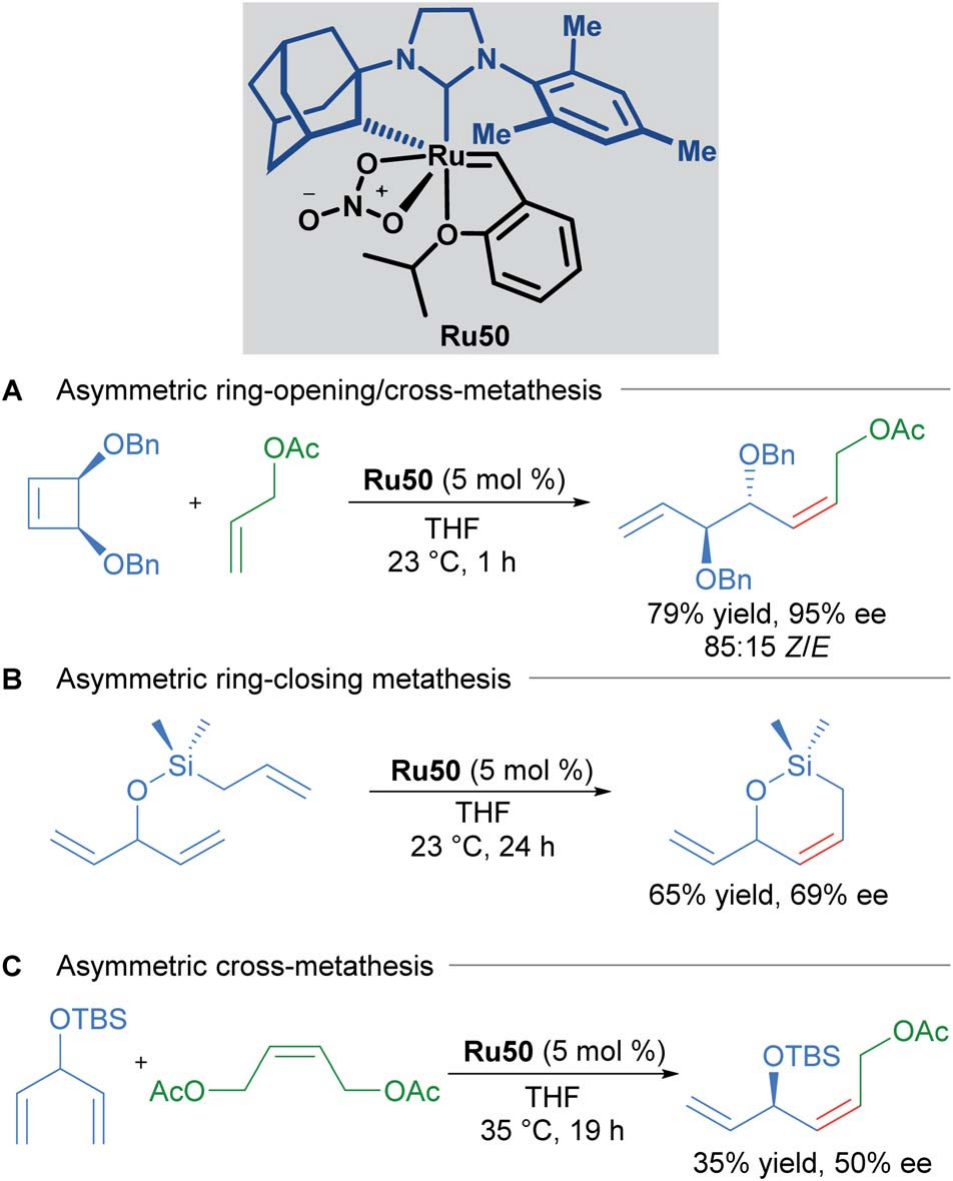
Enantioselective olefin metathesis with cyclometallated ruthenium complexes.

The AROCM of substituted norbornene derivatives with various terminal olefins afforded products with excellent *Z*-selectivity and enantioselectivity. In further exploration of this asymmetric transformation, Grubbs and co-workers extended the scope of the AROCM to other norbornenes with cyclobutenes. The asymmetric ring-closing metathesis of trienes, along with an example of asymmetric cross metathesis to forge allylic stereocentres was developed (Scheme 29).^58^

**Scheme 30:**
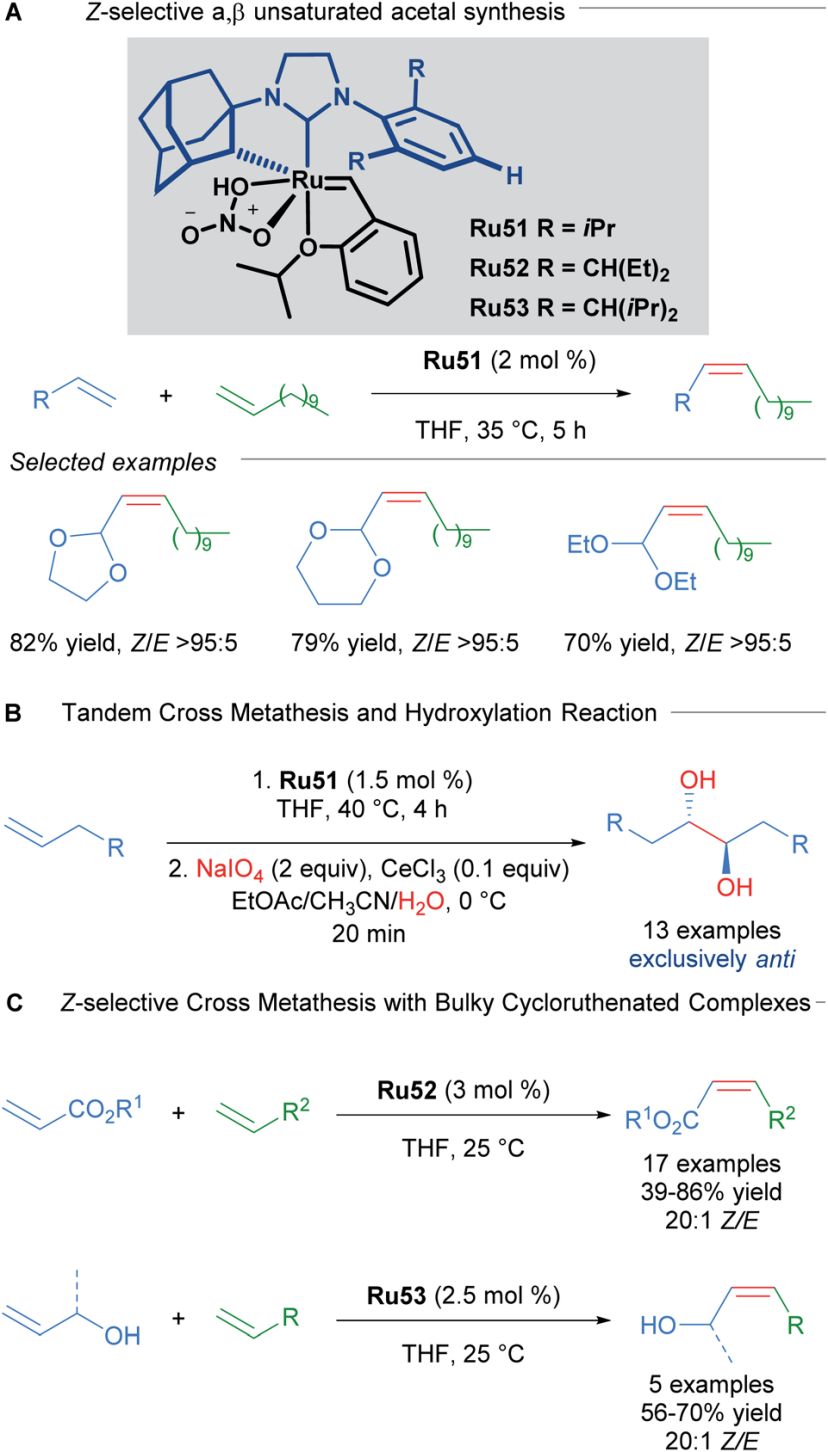
*Z*-selective α,β-unsaturated acetal synthesis and tandem cross metathesis-hydroxylation.

In another example that illustrates the effectiveness of the nitrato-based ruthenium catalysts, Grubbs described the highly *Z*-selective cross metathesis reaction of different terminal olefins, synthesising for the first time, *Z*-α,β-unsaturated acetals with high yields and almost complete *Z*-selectivity (Scheme 30A).^59^ In 2015, Grubbs and co-workers also described the tandem *Z*-selective cross-metathesis and dihydroxylation reactions of olefins to obtain anti-1,2-diols (Scheme 30B).^60^ First, the homo- and hetero- cross metathesis reaction was conducted under static vacuum to remove ethylene and minimise catalyst decomposition. After completion of the *Z*-selective metathesis reaction, the crude was treated with a solution of NaIO_4_ and CeCl_3_ in a mixture of EtOAc : MeCN : H_2_O, affording the desired 1,2-diols with complete anti selectivity. This complete anti selectivity was possible due to the high *Z*-selectivity in the first step. Recently, the authors have developed related ruthenium catalysts Ru52 and Ru53, where increased steric bulk on the aromatic ring afforded excellent *Z*-selectivity in the cross-metathesis of acrylates and allylic alcohols (Scheme 30C).^61^

**Scheme 31:**
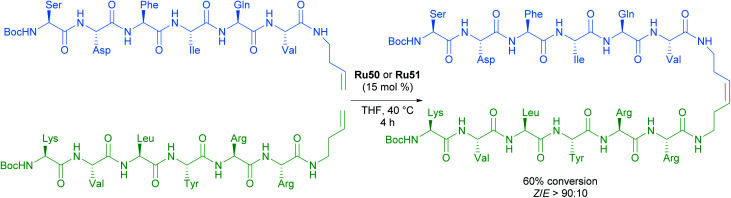
Selected example of cross metathesis polypeptides.

The versatility and efficiency of these NO_2_ derived complexes was also demonstrated with the use of more challenging substrates such as peptides (Scheme 31).^62^ The homodimerisation, cross metathesis and ring-closing metathesis of different peptides were all reported to proceed with high *Z*-selectivity.

Cycloruthenated complexes developed by Grubbs have proven to be extremely effective in *Z*-selective metathesis reactions. In particular, introduction of a cyclometallated adamantyl NHC and a NO_2_ ligand has delivered robust and highly reactive complexes, capable of catalysing metathesis reactions with a wide variety of olefinic substrates. The development of these catalysts has led to excellent results independent of the substitution patterns in the alkene.

## 5. Transfer hydrogenation

The hydrogenation of organic compounds is a fundamental process with widespread industrial applications. Two conventional methods of hydrogenation are direct hydrogenation using H_2_ gas and transfer hydrogenation using alternative hydrogen sources. Transfer hydrogenations are attractive for industrial applications, as high-pressures of H_2_ can be avoided and comparatively green reaction conditions can be used. Many ruthenium complexes catalyse transfer hydrogenation, and ruthenium hydrides are often the active species for the reduction of organic substrates *via* hydrogen transfer. Isopropanol is commonly employed as both the hydrogen source and solvent for the formation of metal hydrides, which shifts this reversible transformation in favour of product formation. This section of the review will be focusing on pincer complexes and related cyclometallated species containing a C–Ru bond, emphasising their usage in transfer hydrogenation applications.

**Scheme 32:**
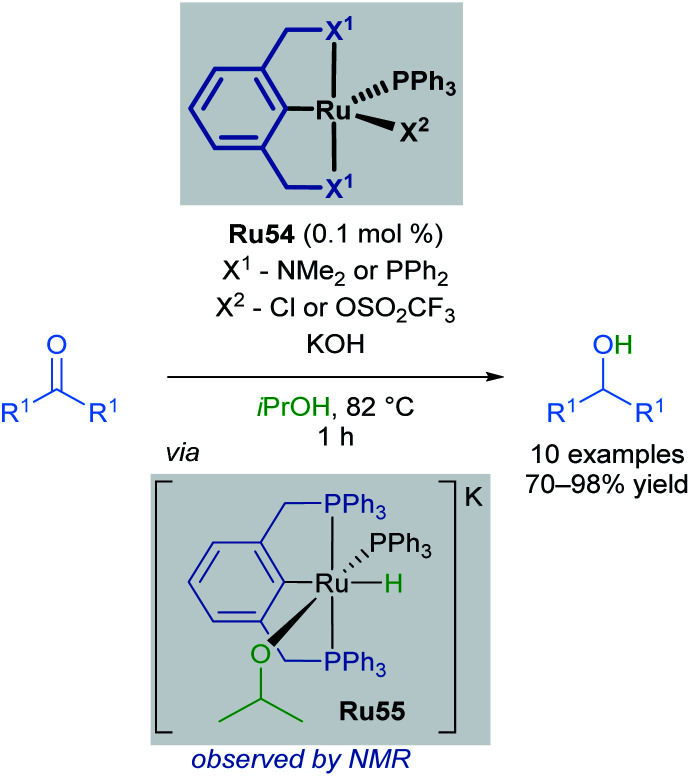
First reported pincer catalysed transfer hydrogenation.

Pincer complexes are widely used and easily tuneable catalysts that offer great control over steric and electronic-properties conferred upon the central metal atom. Complexes that contain a C–M bond are particularly useful, as this strong bond grants excellent thermal stability and prevents decomposition on heating. The first application of ruthenium pincer complexes in transfer hydrogenation reactions was reported by van Koten in 2000 (Scheme 32).^63^ Pincer complexes containing a C–M bond had been successfully used in various transformations including, but not limited to: dehydrogenation,^64^ aldol reactions,^65,66^ and Heck reactions.^67^ Both alkyl and aryl bearing ketones were reduced with high conversions and turnover frequencies (up to 27 000 h^−1^), improving upon previous reports which made use of monodentate ruthenium complexes. The authors also report the first spectroscopic observation of the corresponding Ru–H complex Ru55, generated upon refluxing Ru54 in iPrOH/KOH, confirming that iPrOH serves as the hydrogen source for the formation of this complex.

**Scheme 33:**
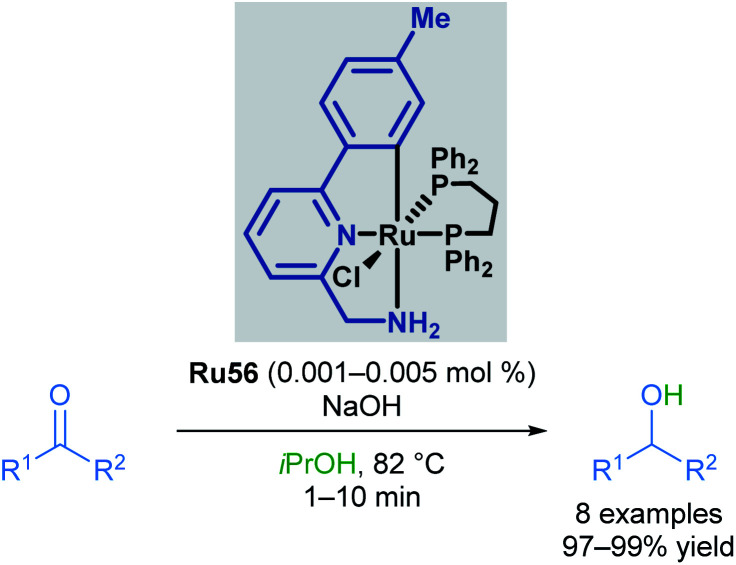
Baratta's transfer hydrogenation of ketones.

In the following years, Baratta and co-workers reported several Ru(ii) pincer complexes for the transfer hydrogenation of various functional groups. The first report in 2005 described the reduction of ketones to the corresponding alcohols in up to 99% yield (Scheme 33).^68^ The reaction catalysed by Ru56 was successful for aryl and alkyl ketones, giving high yields; however, bulky ketone groups could not be reduced. The complex could also be generated *in situ* without loss of reactivity. The Ru–H species was also observed by NMR and IR spectroscopy upon mixing the catalyst with NaOiPr in a solution of iPrOH/toluene.

**Scheme 34:**
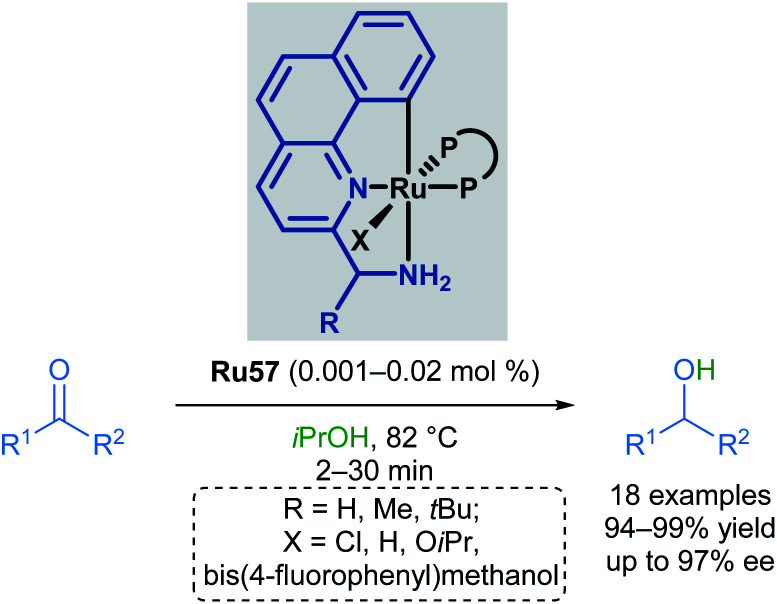
Benzo[*h*]quinoline pincer catalyst in transfer hydrogenation.

Baratta later reported the transfer hydrogenation reaction of several carbonyl-containing functional groups in 2008, this time making use of a ruthenium pincer complex with benzo[*h*]quinoline-based ligands (Scheme 34).^69^ A number of CNN ruthenium pincer complexes were prepared and their catalytic activities examined. Each of the catalysts reported had similar reactivity, furnishing the desired alcohol in over 94% yield with improved TOF. By switching from dppb to bulky, chiral bisphosphines such as (*R*,*S*_p_)- or (*S*,*R*_p_)-Josiphos, (*S*,*S*)-Skewphos or (*S*)-MeO-BIPHEP, enantioselectivities up to 97% ee could be achieved.

**Scheme 35:**
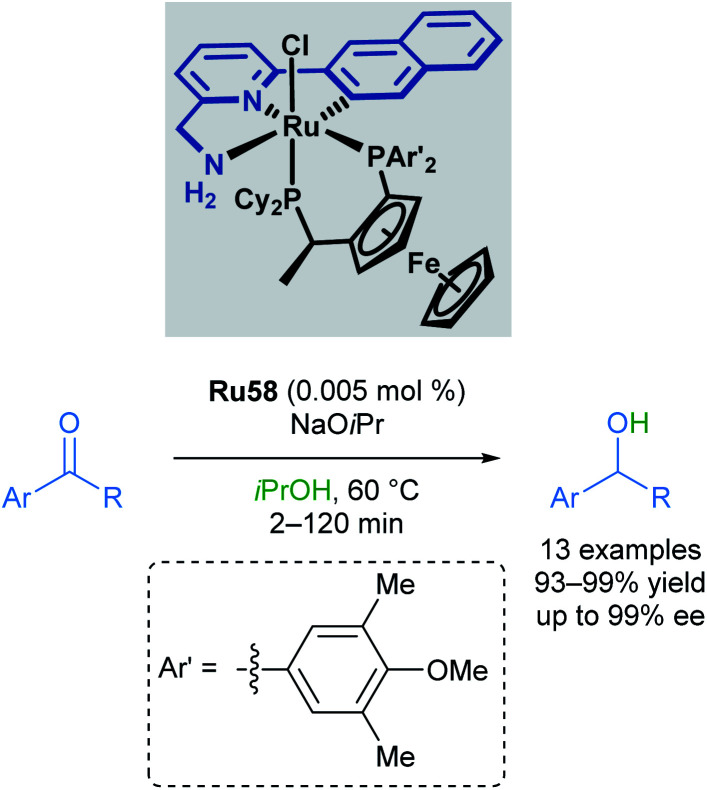
Chiral ruthenium pincer complex for asymmetric transfer hydrogenation.

In 2010, Baratta reported another chiral ruthenium pincer complex Ru58 for the asymmetric transfer hydrogenation of ketones (Scheme 35).^70^*Ortho*- and *meta*-substitution on the aromatic ketone, as well as heteroarenes, were well-tolerated, giving excellent yields (up to 99%) and enantioselectivities (up to 99%). Attempted reductions of methyl ketones were less successful, leading to significantly lower yields (10–20%) and racemic products.

**Scheme 36:**
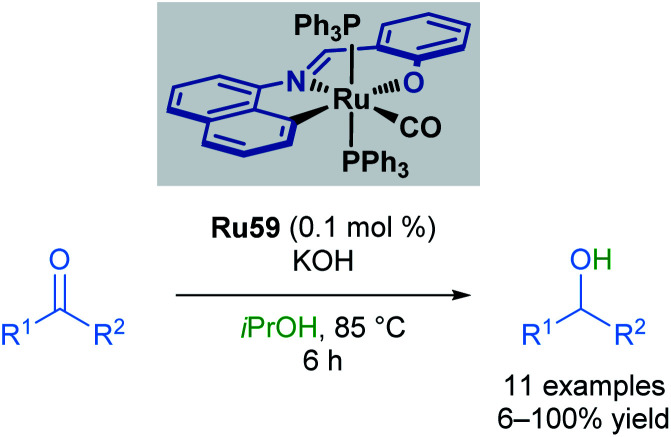
ONC pincer complex in transfer hydrogenation.

In 2014, Bhattacharya and co-workers reported the use of a ruthenium ONC pincer catalyst Ru59 in the transfer hydrogenation of aldehydes and ketones (Scheme 36).^71^ These reductions were conducted with low catalyst loadings (0.1 mol%) and a reaction time of 6 hours. Several examples proceeded in comparable yields to the reduction of aromatic ketones and aldehydes. Substituents at the *para* positions were well tolerated (up to quantitative yields), while substituents at the *ortho* position decreased yields (21% yield). Heteroaromatic rings were not tolerated under these reaction conditions, with pyridine and pyrrole-containing aldehydes being unreactive. This was attributed to catalyst inhibition caused by the strong binding ability of these heterocycles to the metal centre. Aliphatic ketones also furnished the desired alcohols, albeit in lower yields (<50%).

**Scheme 37:**
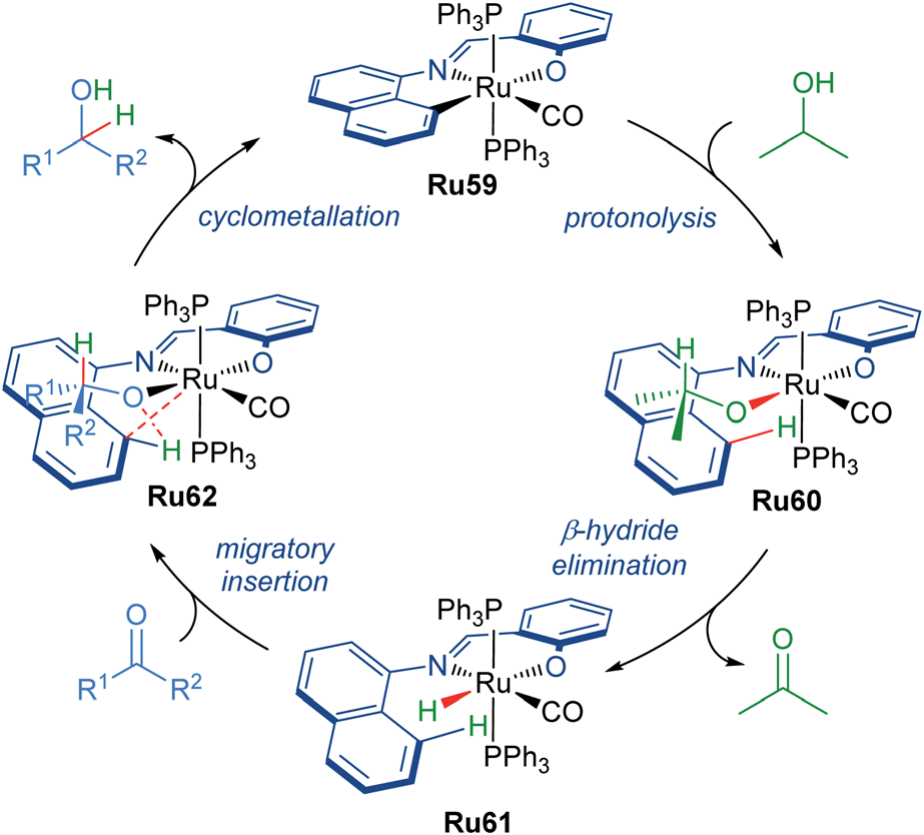
Proposed mechanism with cyclometallated ruthenium pincer complex.

The proposed mechanism for the transformation starts with ruthenium complex Ru59 as the catalyst precursor (Scheme 37). The C–Ru bond then undergoes protonolysis with iPrOH to form intermediate Ru60. This is followed by the β-hydride elimination of the isopropoxide ligand to afford complex Ru61. The carbonyl group can then insert into the Ru–H bond generating intermediate Ru62. Elimination of the product alcohol completes the catalytic cycle, reforming intermediate Ru59.

**Scheme 38:**
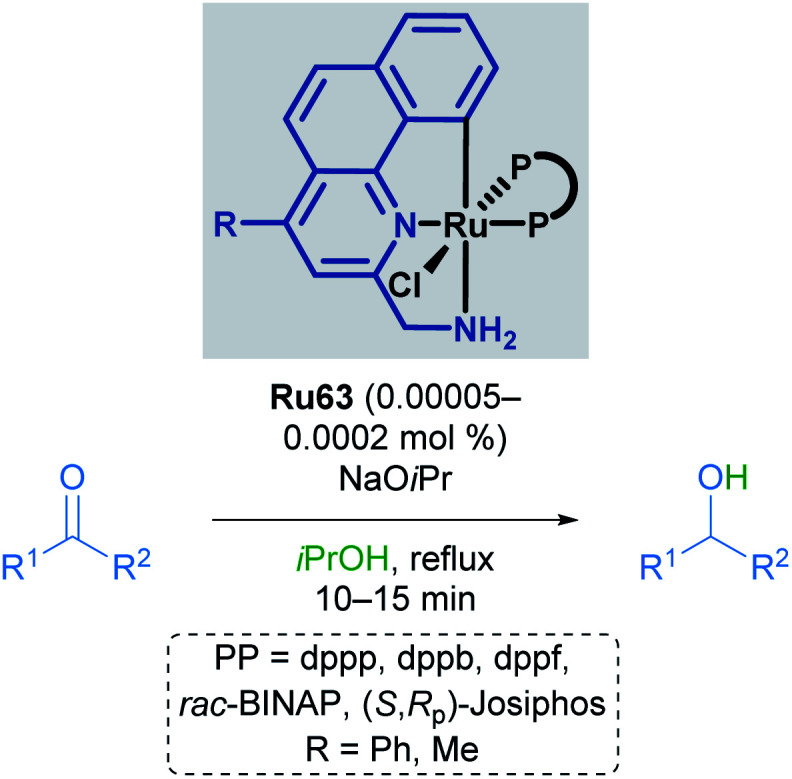
Improved transfer hydrogenation conditions.

Baratta and co-workers continued to expand their library of complexes, describing several additional cyclometallated ruthenium complexes that could perform transfer hydrogenation (Scheme 38).^72^ A notable advancement made was the transformation of bulky ketones to their corresponding alcohols, as previously reported catalytic methods have struggled in this area. It is worth noting that these catalysts also perform the standard hydrogenation reaction of various ketones at low pressures of hydrogen (up to 96% yield).

**Scheme 39:**
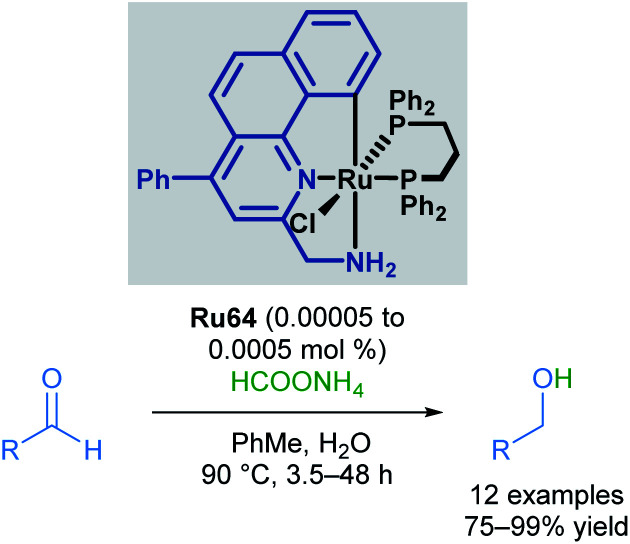
Transfer hydrogenation of aldehydes.

Baratta also reported the chemoselective reduction of aldehydes catalysed by a ruthenium pincer complex, a difficult substrate for transfer hydrogenation due to multiple competing side reactions (Scheme 39).^73^ The desired alcohols were synthesised from the reduction of aliphatic, substituted aromatic, heteroaromatic and conjugated aldehydes, employing inexpensive ammonium formate (HCOONH_4_) as the hydrogen source. Under these conditions, aldehydes were reduced successfully, with no amination or condensation side products being observed. Nitro, nitrile and alkene groups were also tolerated under these reaction conditions, without competing reduction of these groups or catalyst poisoning. This reaction benefits from operational simplicity, with the potential for industrial applications.

**Scheme 40:**
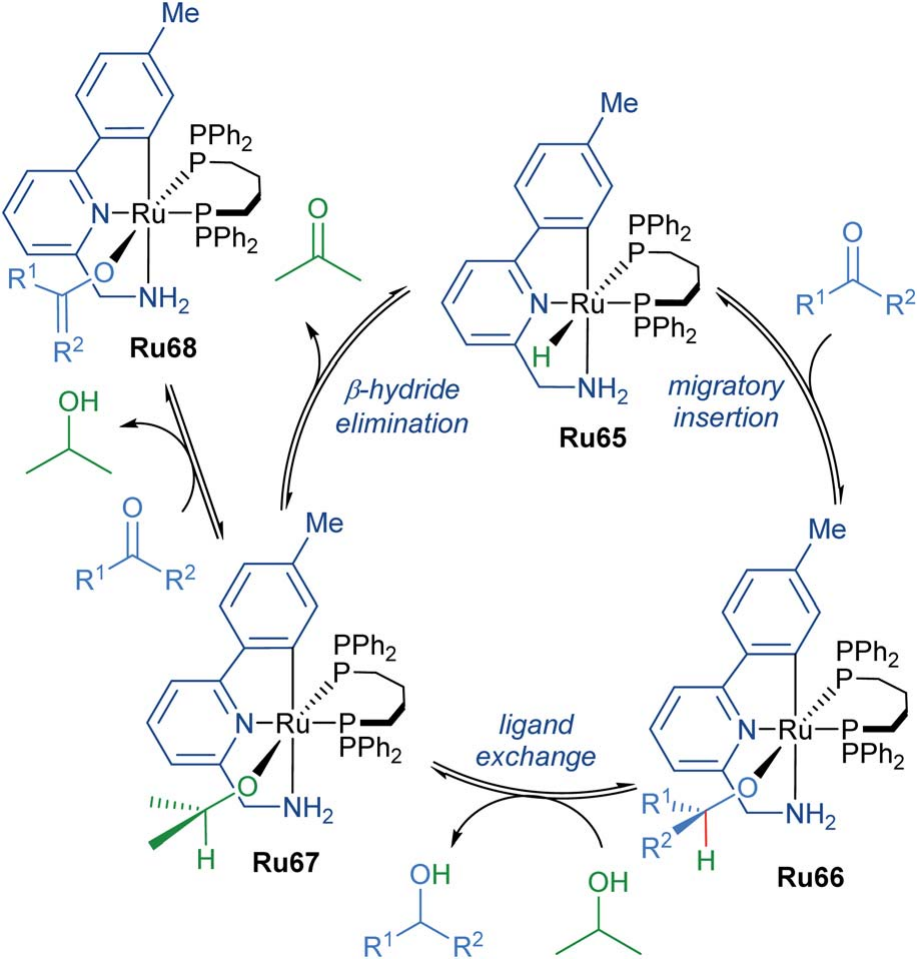
Transfer hydrogenation by orthometallated Ru–NHC complexes.

The mechanism of ketone transfer hydrogenation by cyclometalated ruthenium complexes was examined by Waymouth in 2017 (Scheme 40).^74^ Metal hydride Ru65 reacts reversibly with the ketone substrate in the first step to generate a ruthenium alkoxide Ru66. Alkoxide exchange leads to the formation of ruthenium isopropoxide Ru67, which is a suspected resting state when isopropanol is present in vast excess. Additionally, an off-path equilibrium leads to the formation of ruthenium enolate Ru68, the formation of which is supported by a decrease in reaction rate at high ketone concentrations.

**Scheme 41:**
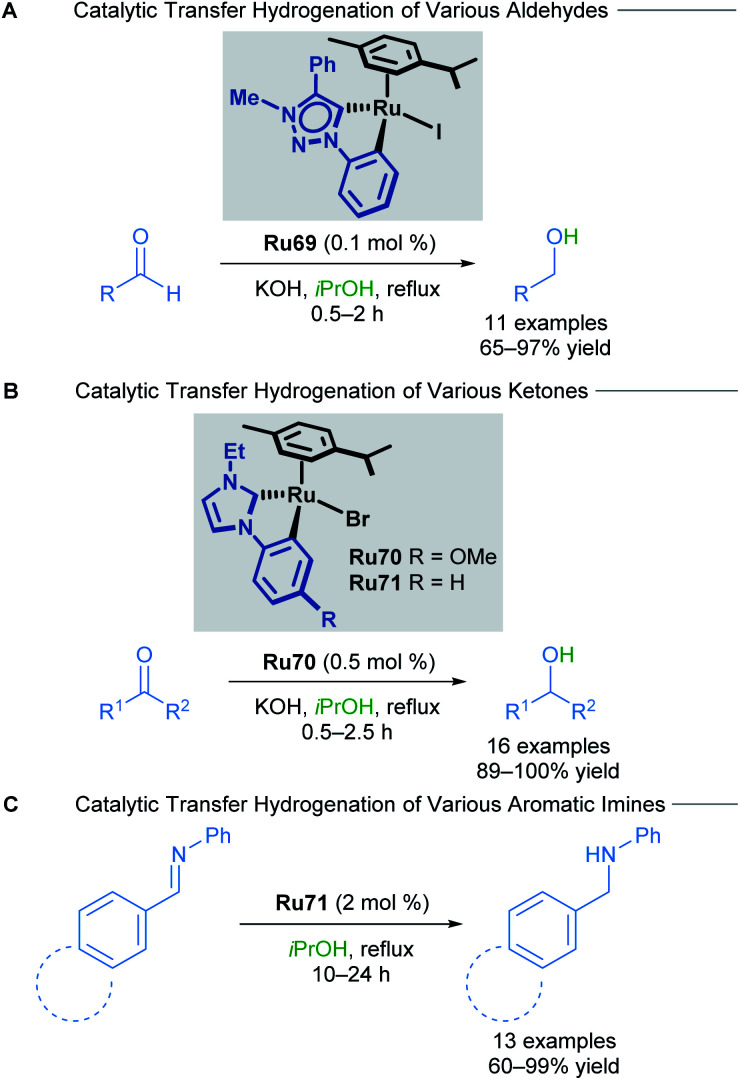
Effect of ancillary ligand in transfer hydrogenation of unsaturated compounds.

In 2018, the Rit group reported the reduction of aldehydes, ketones and imines using orthometallated ruthenium–NHC complexes Ru69–Ru71 to achieve transfer hydrogenation (Scheme 41).^75^ Aromatic ketones were reduced in excellent yields, with both electron-rich and electron-deficient groups being well tolerated. Sterically encumbered groups reacted well, as did 2-acetylpyridine, which is known to be a challenging substrate for this type of reaction due to its ability to poison the catalyst. NHC chelation is proposed to prevent catalyst poisoning from occurring. Both electron-rich and electron-poor aromatic and heteroaromatic aldehydes also reacted smoothly under these conditions. Finally, a variety of aromatic imines were reduced to their corresponding primary amines in high yields using catalyst Ru71. Electron-rich and electron-poor aromatics were once again well tolerated, furnishing the desired amines; however, heteroaromatic imines for this transformation were not reported.

**Scheme 42:**
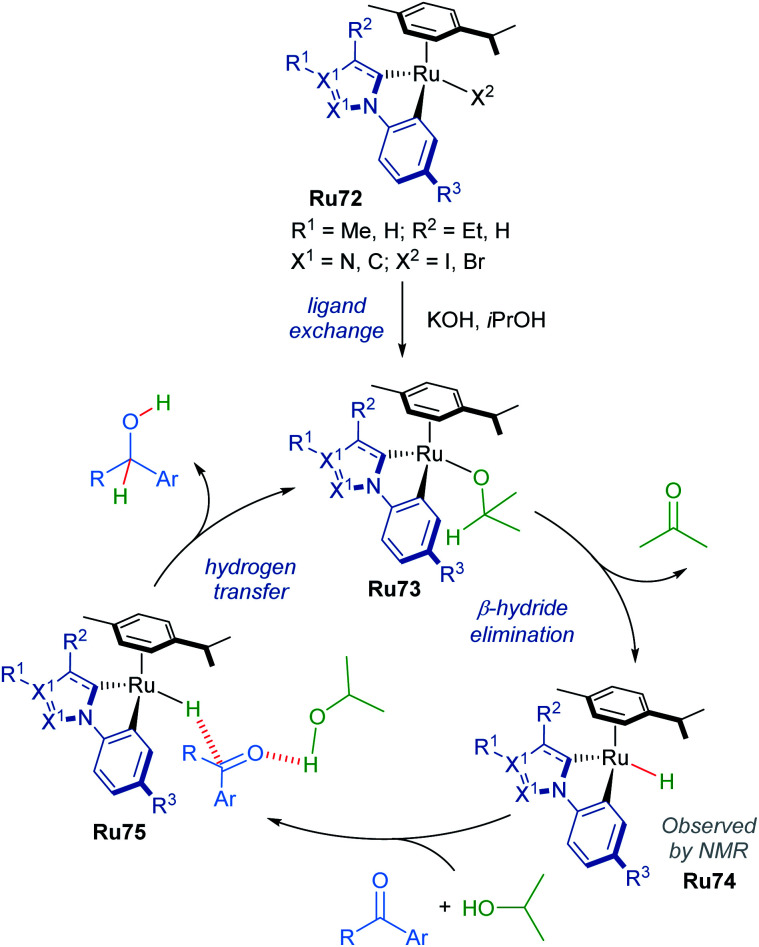
Proposed mechanism for cyclometalated complex.

The mechanism of this reduction was elucidated through NMR spectroscopy (Scheme 42). Excess *p*-cymene led to reduced yields, suggesting that dissociation of a *p*-cymene ligand from the catalyst was likely. Addition of mercury (3 equiv. with respect to the substrate) to the reaction had no effect, suggesting the reaction is homogenous. Furthermore, evidence for the formation of a cyclometallated Ru–H complex is given by ^1^H NMR experiments, with the characteristic signal being detected after refluxing complex Ru72 in KOH/iPrOH.

**Scheme 43:**
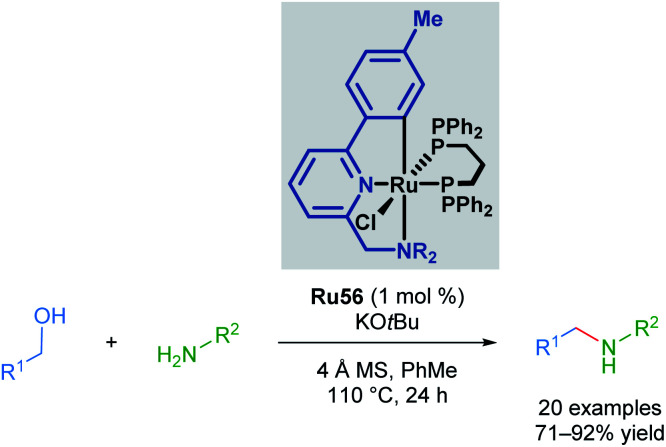
Alkylation of amine with Baratta's catalyst Ru56.

The Martín-Matute group reported a selective mono-alkylation of aromatic and heteroaromatic amines with primary alcohols catalysed by Baratta's readily available Ru(ii) pincer complex Ru56, with no polyalkylation leading to the formation of tertiary amines observed in the presence of excess alcohol (Scheme 43).^76^ This represents an environmentally friendly procedure, as water is produced as the sole by-product. This reaction is thought to proceed *via* the formation and subsequent reduction of an imine by a transient metal hydride. This was the first application of a ruthenium-pincer complex in the reduction of imines. The authors also managed to achieve selective *N*,*N*-dialkylation where two amine functional groups were present in the substrate when 2 equivalents of the alcohol were used. When amines that cannot be oxidised to form an imine were used, no product was observed, suggesting that the reaction proceeds *via* imine formation and subsequent reduction.

**Scheme 44:**
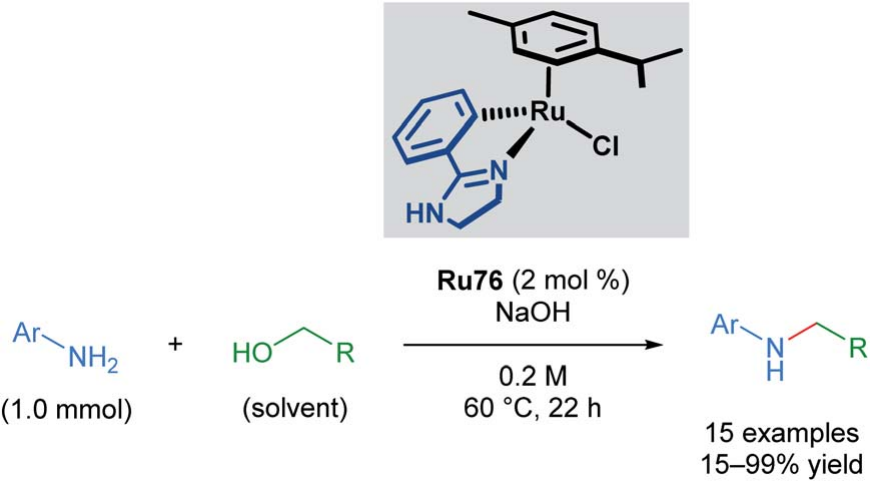
Beller's alkylation of a primary amine with alcohols catalysed by a cycloruthenated phenylimidazoline complex.

Recently, Beller and co-workers applied cyclometallated imidazoline ruthenium complexes in the *N*-methylation of several aromatic anilines (Scheme 44).^77^ This transformation has literature precedent with other catalysts; however, the use of a cyclometallated ruthenium complex allows milder conditions and a less expensive base to be used. Although steric bulk in the *ortho*-position of the aromatic aniline was poorly tolerated, the reaction proceeded with *meta*- and *para*-substituents. An electron-withdrawing nitro group *para*- to the aniline led to reduced yields of 28%; however, no reduction of the nitro group was observed. Electron-rich anilines were transformed in excellent yields. 4-Amido-aniline was also able to undergo transformation without competing amide functionalisation. Application of this method to primary amines was unsuccessful.

**Scheme 45:**
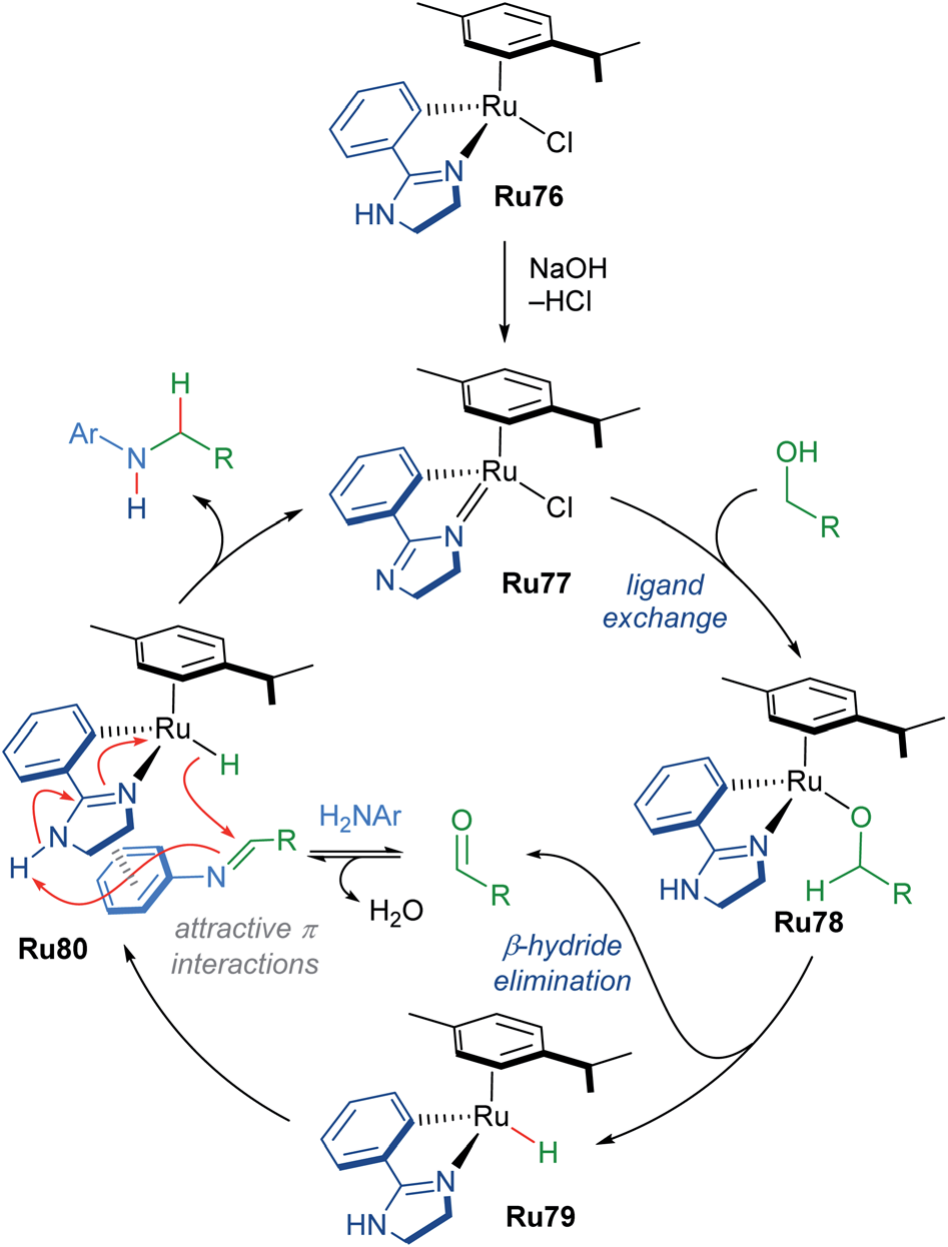
Proposed mechanism for *N*-alkylation of amines with alcohols.

A mechanism for *N*-methylation was proposed by the authors (Scheme 45). The initial step involves reaction of the precatalyst with NaOH, which is supported by ^1^H NMR experiments in which the precatalyst Ru76 was pre-mixed with base in MeOD. Next, the alcohol coordinates to the catalyst to form intermediate Ru78, which undergoes rate-determining β-hydride elimination, as supported by a KIE value of 1.8 measured for MeOH-*d*_3_. This forms a Ru–H species Ru79, which reacts with the generated imine through an associative π-interaction between the two species (Ru80), yielding the desired product. The reaction was insensitive to the presence of mercury, suggesting that homogeneous catalysts are operative in this process.

Readers are directed to a recent review by de Vries for additional and extensive coverage of ruthenacycles in transfer hydrogenation.^78^

## 6. Enantioselective cyclopropanation

**Scheme 46:**
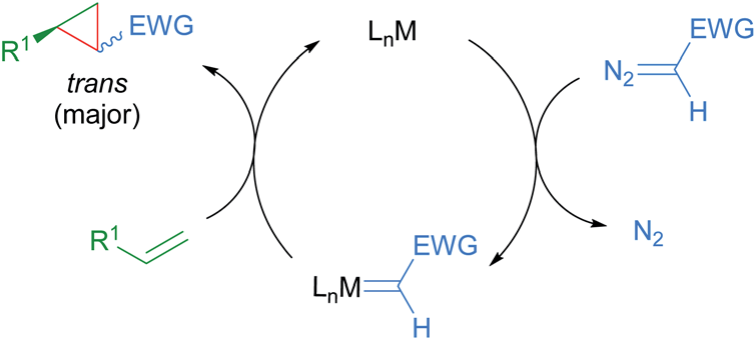
General mechanism for metal-catalysed enantioselective cyclopropanation through diazo decomposition.

The prevalence of cyclopropane-containing natural products, agrochemicals and therapeutic agents has driven the continuous development of new methods for enantioselective cyclopropanation. Specifically, metal-catalysed enantioselective cyclopropanation proceeding through diazo decomposition offers direct and stereocontrolled access to optically active and functionalised cyclopropanes.^79^ Since the seminal report by Noyori in 1966,^80^ several chiral metal complexes have been shown to catalyse this transformation, typically using stabilised diazoacetates as carbene precursors. More recently, ruthenium complexes have emerged as viable catalysts for enantioselective cyclopropanation.^81^ The accepted catalytic cycle proceeds through metal-catalysed decomposition of a diazoalkane with concomitant loss of dinitrogen to form an intermediate metal carbenoid. Subsequent carbene transfer to an alkene provides the cyclopropane product, often resulting in preferential formation of the *trans* diastereomer (Scheme 46). Selected examples from the recent literature on enantioselective cyclopropanations catalysed by cycloruthenated complexes will be presented in this section. Readers are also referred to a perspective by Iwasa for his pioneering work on enantioselective cyclopropanations catalysed by Ru(ii)-phenyloxazoline (Pheox) complexes presented in this section.^82^

**Scheme 47:**
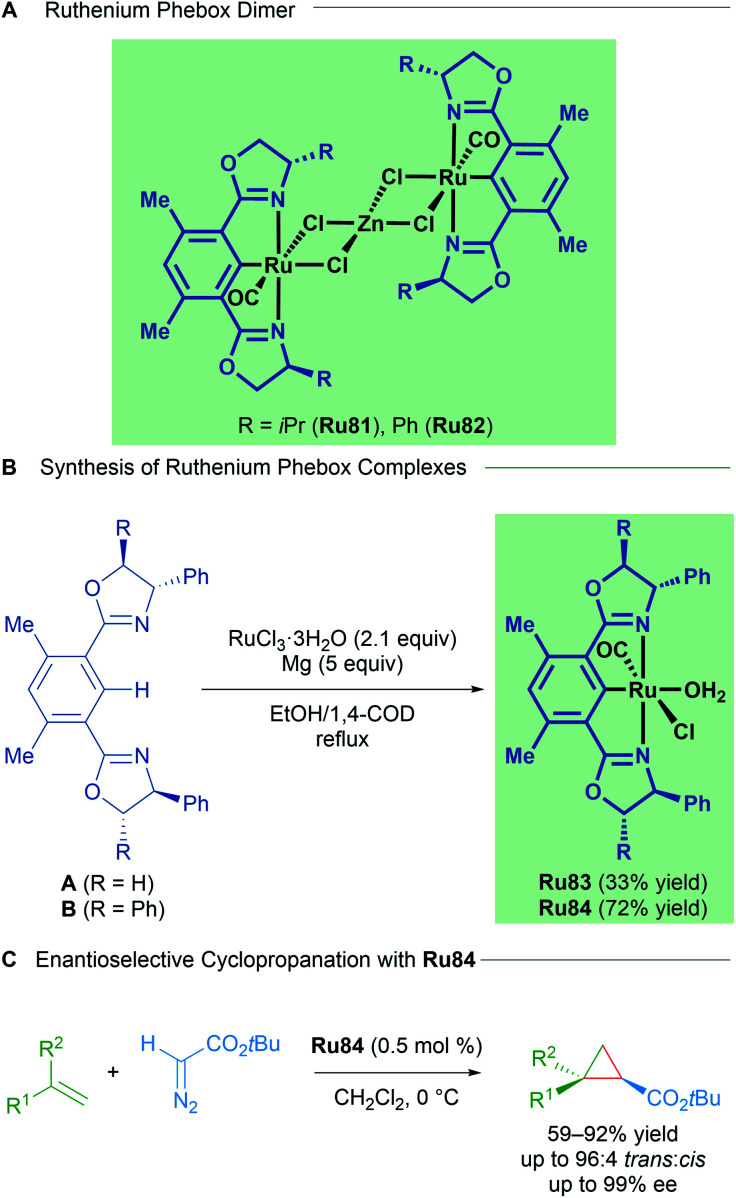
Nishiyama's Ru–Phebox complexes for enantioselective cyclopropanation of alkenes.

Examples of structurally well-defined cyclometallated ruthenium complexes applied to enantioselective cyclopropanation were reported by the group of Nishiyama in 2010.^83^ Substitution of the central pyridine ring in the Pybox ligand for a benzene yields chiral bis(oxazolinyl)phenyl NCN pincer ligands (Phebox). The resulting Ru(ii)–Phebox complexes (Ru81 and Ru82) had previously been synthesised and characterised as heterobimetallic, dinuclear Ru–Phebox units bridged by ZnCl_4_ (Scheme 47). Following this result, the authors synthesised mononuclear aqua complexes Ru83 and Ru84 from the magnesium reduction of RuCl_3_·3H_2_O in an ethanolic solution of 1,4-cyclooctadiene. Each of the complexes (Ru81–Ru84) possess a Ru–C σ-bond, with a single CO ligand assumed to derive from the oxidation of ethanol to acetaldehyde, followed by decarbonylation. Complexes Ru81–Ru84 were effective catalysts for the *trans*-selective enantioselective cyclopropanation of styrene derivatives using bulky *tert*-butyl diazoacetates. The reported enantioselectivities reached 99% in several instances.

**Scheme 48:**
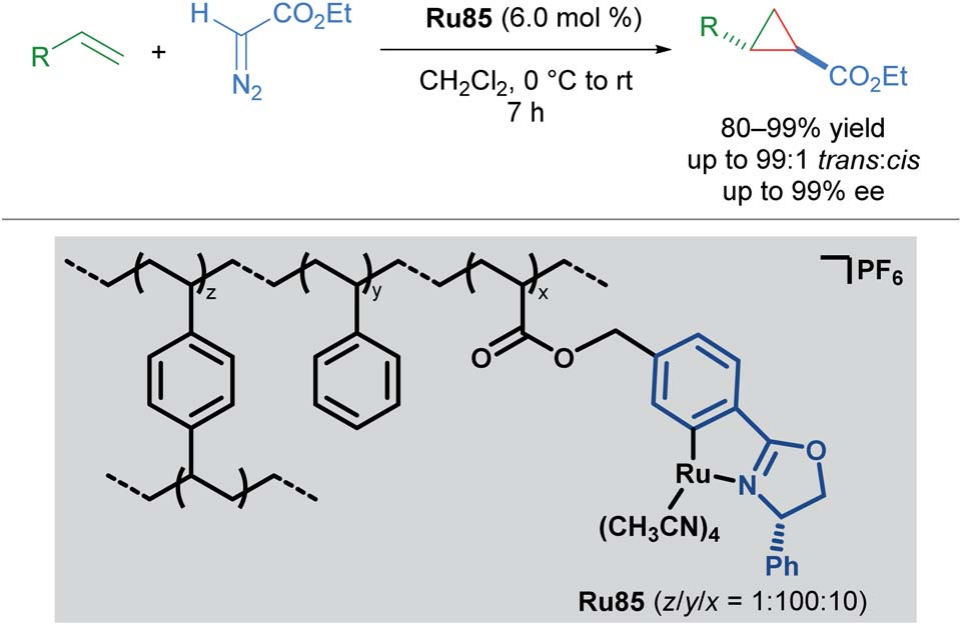
PSCC applied to enantioselective cyclopropanation.

Concurrently, the group of Iwasa reported the use of a polymer-supported, chiral ruthenium–phenyloxazoline complex (Ru–pheox) for enantioselective cyclopropanation in 2010 (Scheme 48).^84^ Polymer-supported chiral catalysts (PSCCs) have been investigated extensively as reusable alternatives to homogeneous catalysts. The authors highlight a new synthetic strategy for the synthesis of a PSCC, wherein a novel Ru(ii) phenyloxazoline complex was synthesised as a monomeric species that underwent efficient crosslinking polymerisation with styrene and 1,4-divinylbenzene in the presence of H_2_O using AIBN as the initiator. The resulting catalyst Ru85 was determined to be highly reactive, and catalysed both inter- and intramolecular enantioselective cyclopropanation with excellent diastereo- and enantioselectivity. The long-term stability over three months and re-useability of the PSCC after ten applications was also demonstrated. Higher reactivity relative to other PSCCs examined was attributed to the greater internal surface area of Ru85 as revealed by scanning electron microscopy. Greater accessibility to the catalyst active site may also account for the higher reactivity observed with Ru(ii)–Pheox catalysts compared to bisoxazoline pincer catalysts in homogeneous cyclopropanation.

**Scheme 49:**
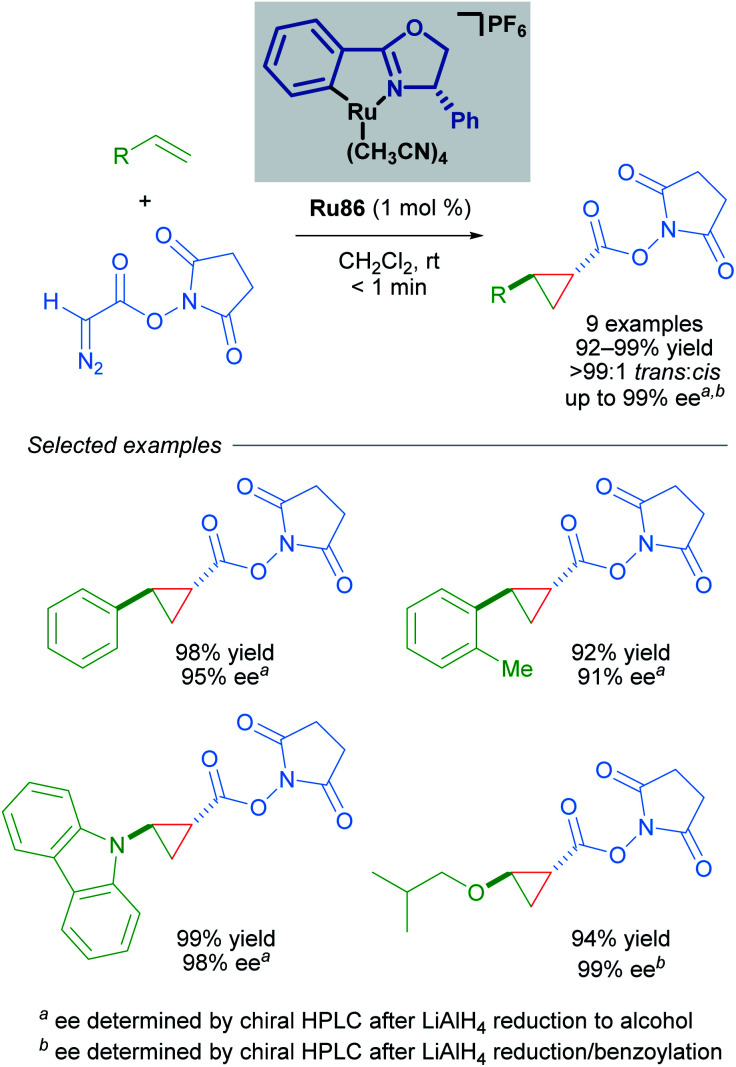
Enantioselective cyclopropanation of styrenes with succinimidyl diazoacetates catalysed by a homogeneous catalyst Ru86.

Building upon this seminal work, the group of Iwasa developed a homogeneous Ru(ii)–Pheox catalyst and demonstrated that a well-defined mononuclear catalyst Ru86 can be synthesised from commercially available starting materials in a few short steps.^85^ Using succinimidyl diazoacetates as carbene precursors, chiral cyclopropanes could be synthesised from various terminal olefins with excellent diastereo- and enantioselectivity under very mild conditions (Scheme 49). In these reactions, succinimidyl chelation is thought to account for enhanced *trans*-diastereoselectivity.

**Scheme 50:**
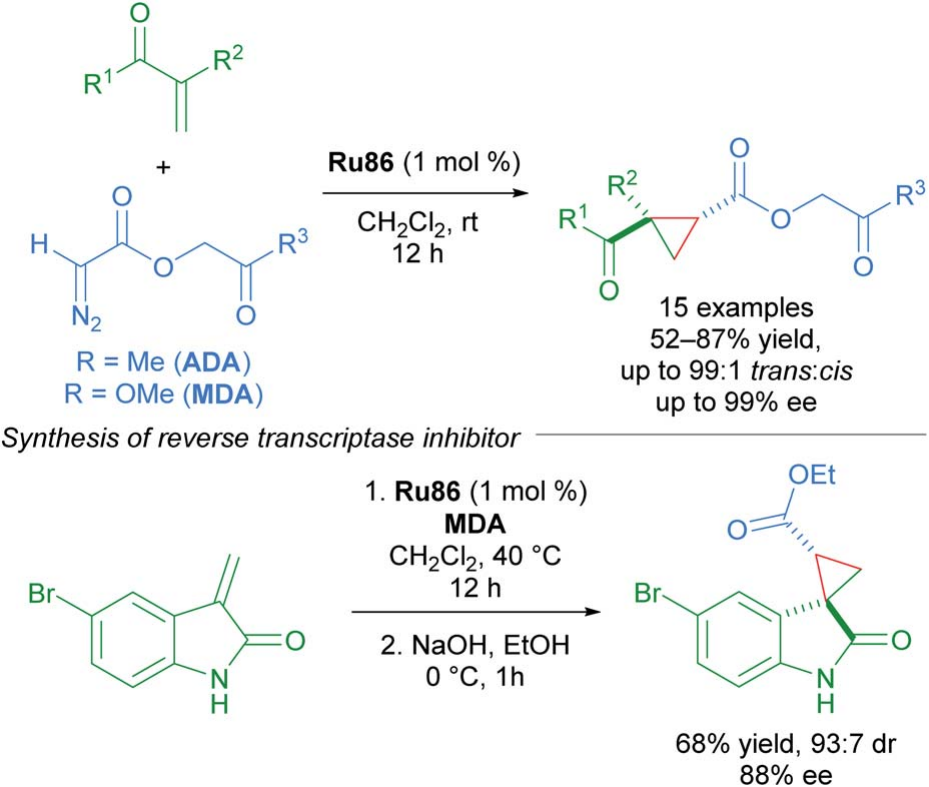
ADA and MDA as carbene precursors applied to the synthesis of an HIV-1 non-nucleoside reverse transcriptase inhibitor.

The enantioselective cyclopropanation of electron-deficient olefins represents a key advance in the chemistry of Ru(ii)-Pheox complexes. The first use of acetonyl diazoacetate (ADA) and methyl (diazoacetoxy)acetate (MDA) as carbene sources, with MDA being better in some cases. In general, the dicarbonyl cyclopropane products can be accessed in high yields with excellent diastereoselectivity (up to >99 : 1) and enantioselectivity (up to 99% ee). A key application was towards the synthesis of an HIV-1 non-nucleoside reverse transcriptase inhibitor (Scheme 50).^86^

**Scheme 51:**
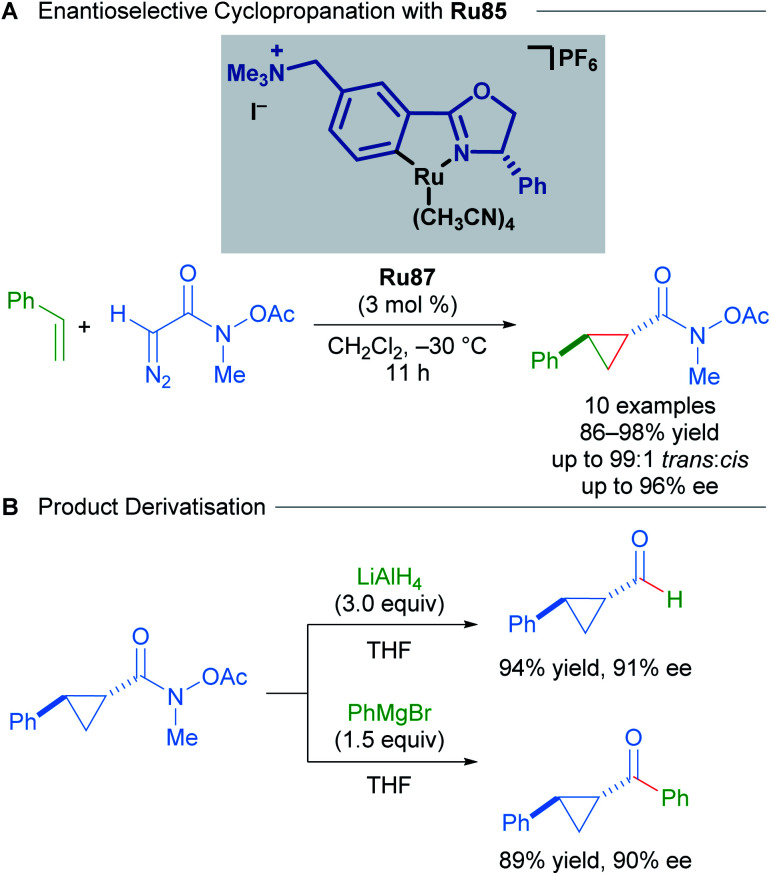
Enantioselective cyclopropanation with Ru87 and subsequent product derivatisation.

In 2016, a further development in catalyst design came in the form of incorporating an ammonium group into the Ru(ii)–Pheox catalysts (Scheme 51).^87^ The quaternary ammonium group affords catalysts with higher reactivity and stereoselectivity. The Ru87 catalyst expanded the scope to include diazo Weinreb amides as coupling partners. The synthetic utility of Weinreb amides was demonstrated through derivatisation experiments.

**Scheme 52:**
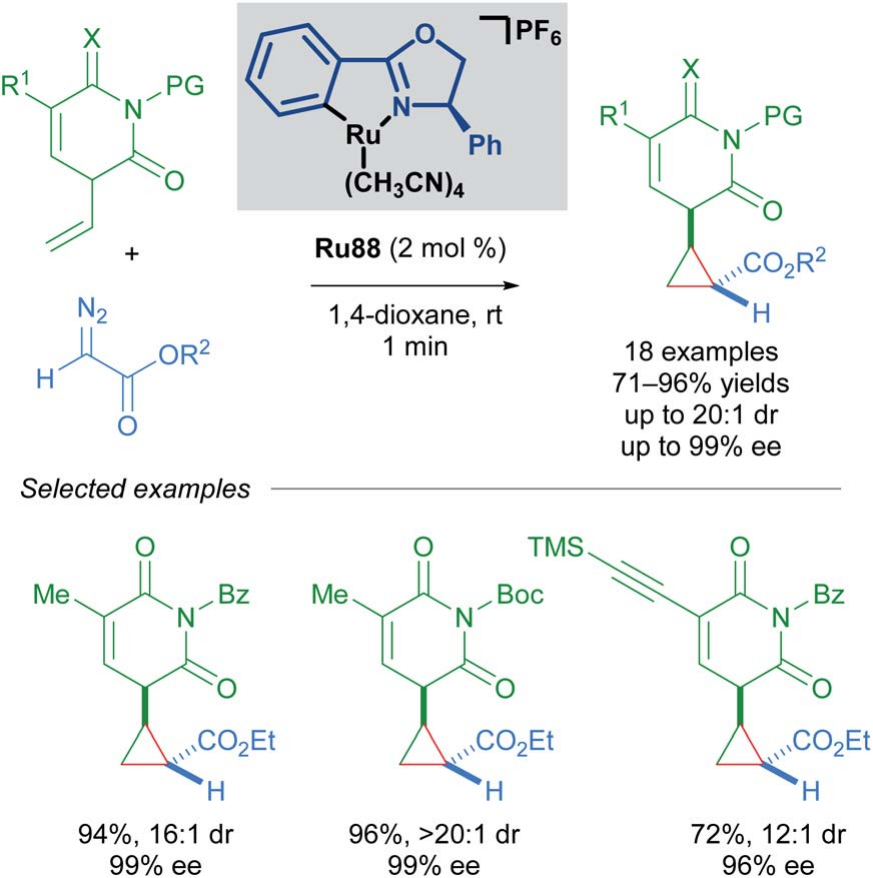
Guo's enantioselective cyclopropanation of pyrimidine nucleoside analogues.

In 2018, the group of Guo has also utilised Ru(ii)–Pheox complex Ru88 in the synthesis of chiral cyclopropyl pyrimidine nucleoside analogues in good yields and enantioselectivities using α-diazoesters as coupling partners (Scheme 52).^88^ The scalability of this method was demonstrated with a gram-scale experiment. Notably, this transformation was rapid and complete within a minute as judged by the cessation of N_2_ evolution.

**Scheme 53:**
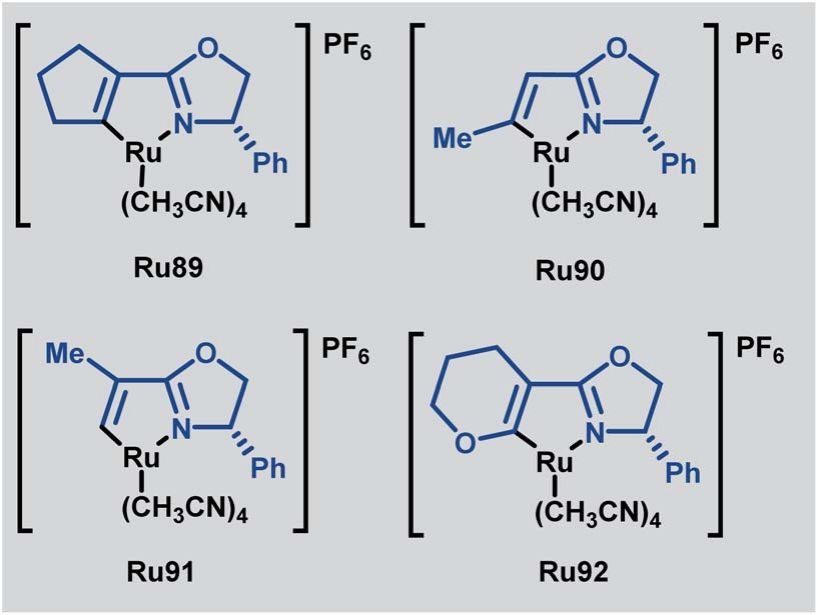
Ruthenium alkenyl oxazoline complexes for enantioselective cyclopropanation.

In 2020, a related series of cationic ruthenium complexes containing Ru–C(sp^2^)–olefin bonds were synthesised through C–H activation of alkenyl oxazoline ligands and applied to enantioselective cyclopropanation (Scheme 53).^89^ Of the newly synthesised complexes (Ru89–Ru92), Ru91 possessed superior reactivity to the standard phenyl-substituted catalyst. The absence of a geminal substituent was found to afford the best enantioselectivities for a range of *trans*-allylic diazoacetates in this transformation.

**Scheme 54:**
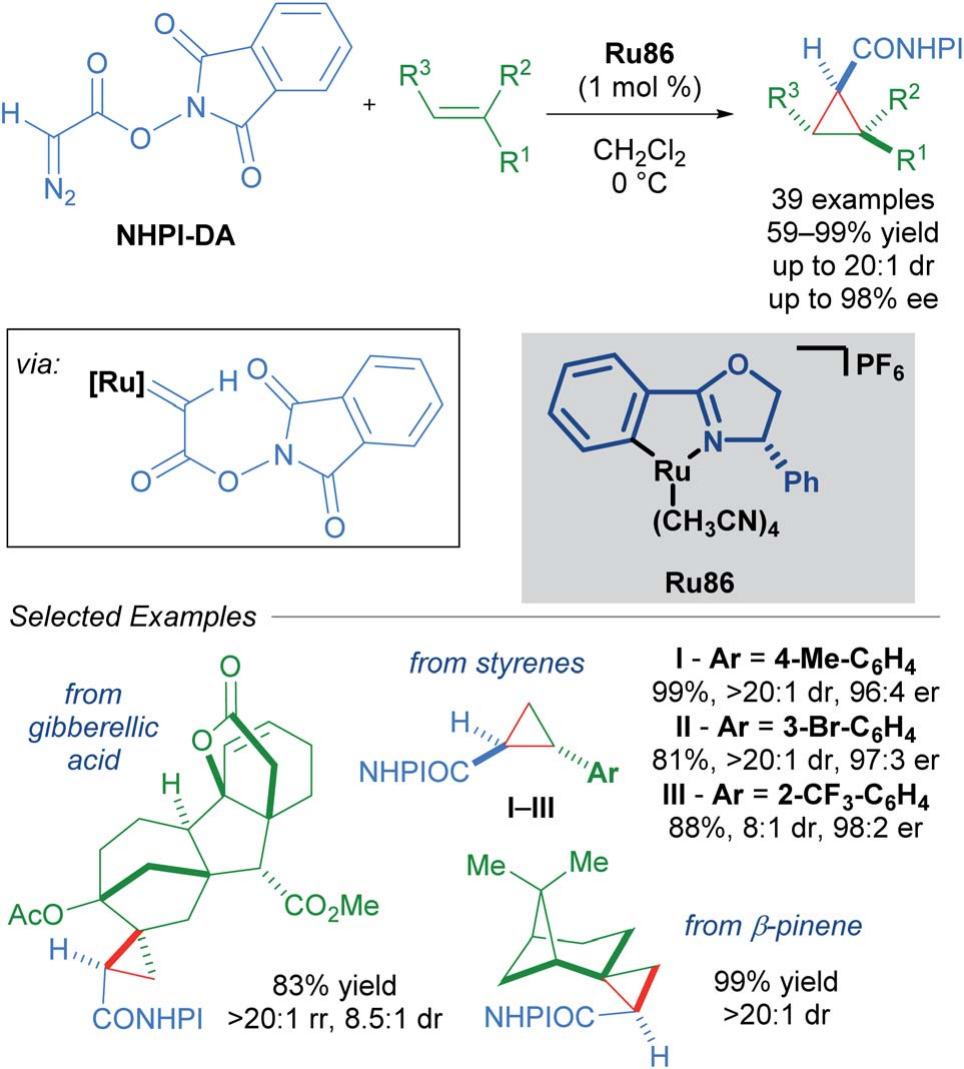
Redox-active carbenes in enantioselective cyclopropanation.

An impressive breakthrough in late-stage diversification was realised by using redox-active carbene precursors, *N*-hydroxyphthalimidoyl diazoacetate (NHPI-DA) reagents. NHPI-DA are crystalline solids that can be synthesised on gram scale and remain bench-stable for several months.^90^ The principal advantage of these reagents lies in the dual capacity of NHPI esters to act as acyl electrophiles and radical precursors. These reagents are equivalent to functionalised carbene transfer reagents. By optimising a single enantioselective cyclopropanation and leveraging the reactivity of the NHPI ester, several new functionalised cyclopropanes could be synthesised in a divergent manner. Initially, commonly used rhodium, copper and palladium catalysts performed poorly when using NHPI-DA as the carbene precursor. Further screening revealed Iwasa's electron-rich cycloruthenated complex Ru86 as a highly reactive catalyst that gave excellent results (Scheme 54).

**Scheme 55:**
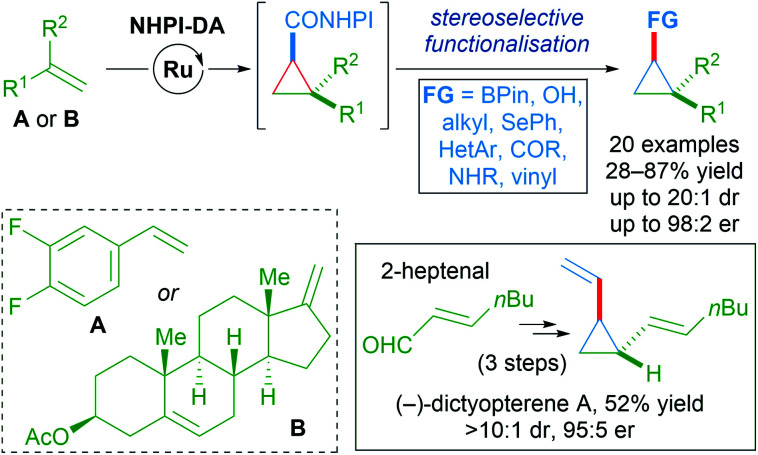
Stereoselective functionalisation of redox-active cyclopropanes A or B.

A wide variety of functional groups could be appended to challenging alkene substrates *via* stepwise enantioselective cyclopropanation followed by stereoselective functionalisation of the redox-active intermediate. A three-step synthesis of (−)-dictyopterene A was also made possible with these reagents (Scheme 55).^90^

## 7. Oxidative cyclisation and cycloaddition

**Scheme 56:**
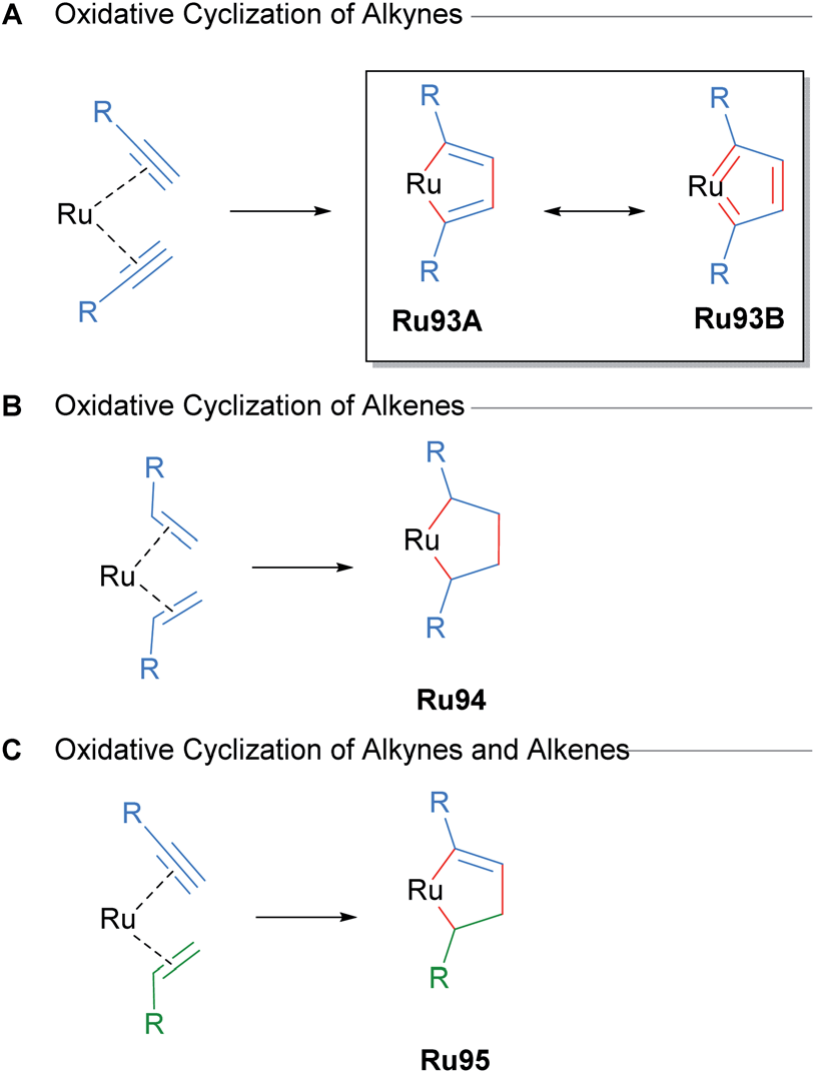
Oxidative cyclisation of unsaturated molecules with ruthenium catalysts.

Metal-catalysed cycloadditions are excellent methods for the synthesis of substituted aromatic compounds. Oxidative cyclometallation of alkynes with ruthenium catalysts has historically yielded a wealth of new ruthenacycle complexes. Typically, low valent, electron-rich sources of ruthenium (*e.g.* CpRuX) can react with two equivalents of alkyne in a concerted fashion to form ruthenacycle intermediates with concomitant oxidation of the metal centre (Scheme 56). Subsequent reactions of these ruthenacycle intermediates furnishes functionalised aromatic products. In many instances, these ruthenacycles have proven to be stable enough for isolation. Indeed, a common intermediate for many of these processes is a ruthenacyclopentatriene biscarbene species Ru93, which was first reported in 1986 by Singleton (Scheme 56A).^91^ Similarly, saturated ruthenacycles can arise from the oxidative cyclisation of alkenes, producing ruthenacyclopentanes Ru94 (Scheme 56B). Oxidative cyclisation between alkynes and alkenes are also known, and these presumably proceed *via* intermediates such as Ru95 (Scheme 56C).^81^ This section will focus on reports of catalytic cycloaddition processes that involve ruthenacycles as precatalysts or intermediates. Readers are also directed to earlier reviews by Trost^11*a*^ and Kirchner^11*b*^ for additional examples.

**Scheme 57:**
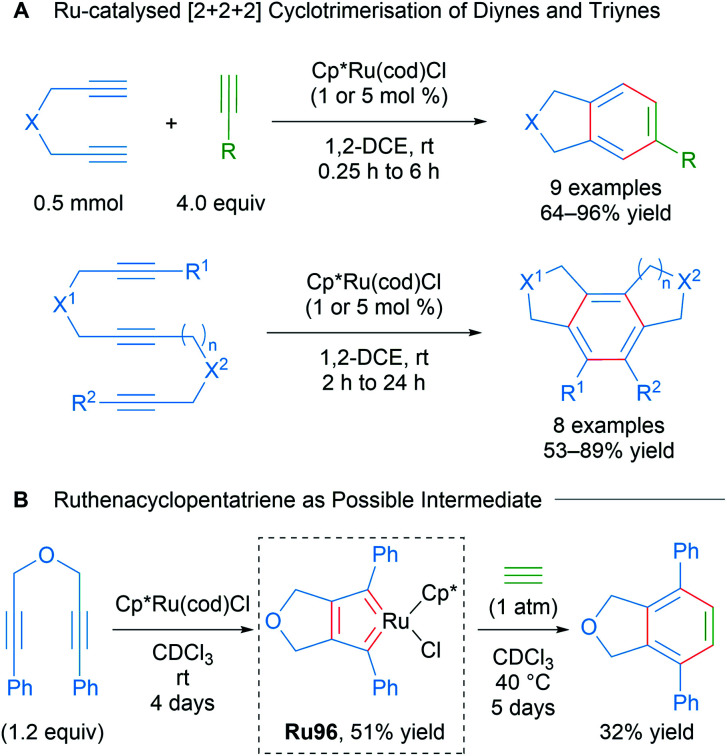
Itoh's ruthenium-catalysed intramolecular cyclotrimerisation of diynes and triynes and possible biscarbene intermediate.

In a comprehensive study from 2003, Itoh and co-workers reported a catalytic protocol for the cyclotrimerisation of diynes and triynes catalysed by Cp*Ru(cod)Cl (Scheme 57A).^92^ The electron-rich Cp*RuCl fragment was identified as the active species in this process. A ruthenabicycle complex Ru96 was synthesised through the stoichiometric reaction of Cp*RuCl(cod) with a 1,6-diyne possessing phenyl terminal groups and characterised by XRD as the biscarbene Ru96. The reported cyclotrimerisation reaction proceeded under mild conditions with excellent selectivity and functional group compatibility, which was notably difficult to achieve in the catalytic cyclotrimerisation of alkynes. The intermediacy of the ruthenacycle complex Ru96 was demonstrated in a stoichiometric reaction (Scheme 57B). The authors have also reported various Cp*Ru(cod)Cl-catalysed cyclotrimerisation cascades involving: 1,6-heptadiynes,^93^ dicyanides,^94^ electron-deficient ketones,^95^ isothiocyanates/CS_2_,^96^ and temporary boron-containing tethers.^97,98^

**Scheme 58:**
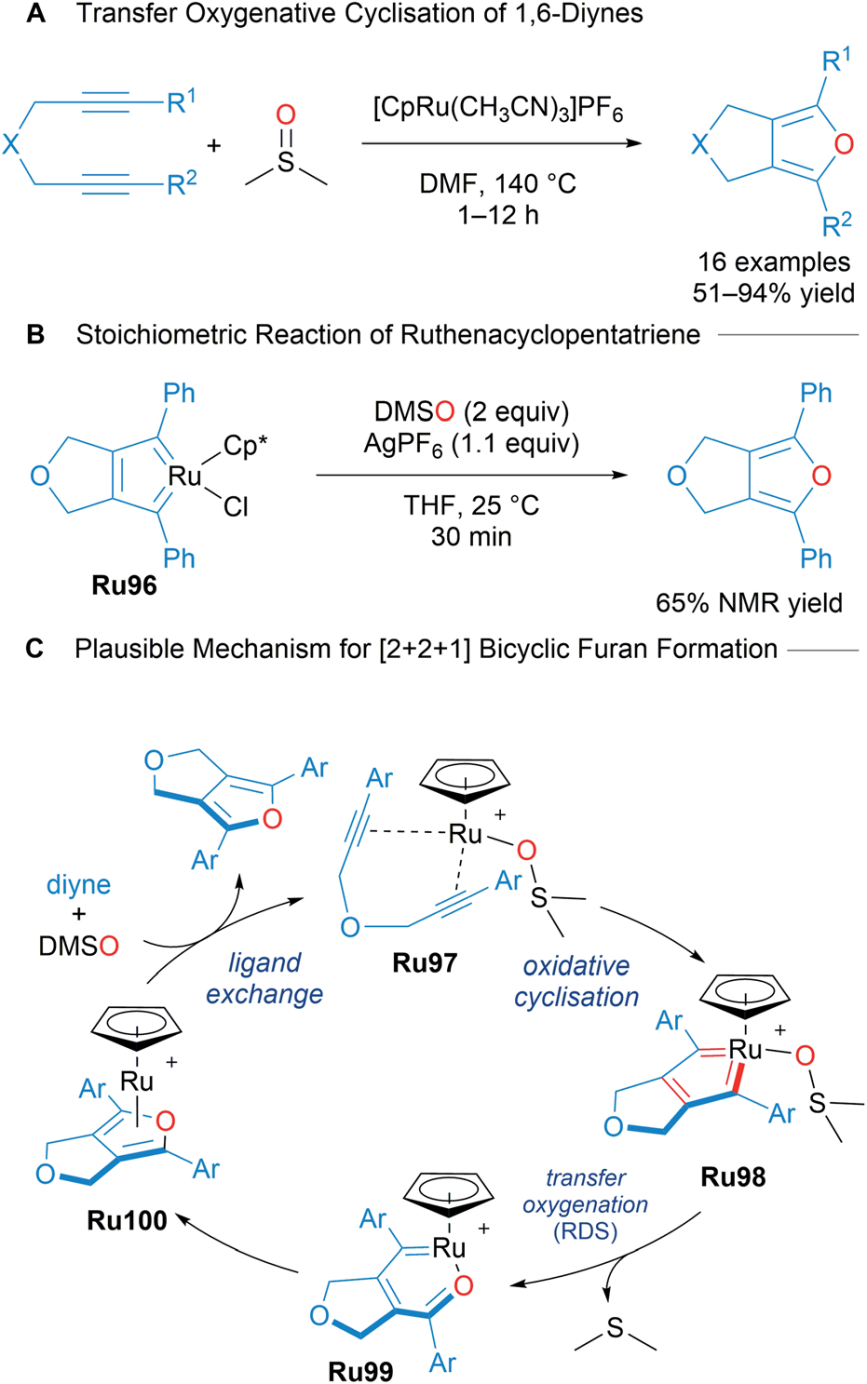
Nishiyama's ruthenium-catalysed transfer oxygenative [2 + 2 + 1] cycloaddition.

Nishiyama reported the catalytic [2 + 2 + 1] cycloaddition of 1,6-diynes for the formation of bicyclic furans using DMSO as the oxygen atom donor (Scheme 58A).^99^ A mechanism for bicyclic furan formation *via*Ru96 was proposed based on both experimental and theoretical studies. An isolated sample of Ru96 was treated with DMSO (2 equiv.) and AgPF_6_ (1.1 equiv.) under the reaction conditions, leading to the formation of a fused furan product, strongly suggestive of its involvement in the catalytic cycle as a key intermediate (Scheme 58B). A plausible mechanism supported by DFT begins with a ruthenacyclopentatriene Ru98 coordinated to DMSO. Oxygen transfer leads to intermediate Ru99 with extrusion of DMS. Ligand exchange with intermediate Ru100 releases the product. This transformation represents a highly atom economical approach towards accessing bicyclic furans through a mechanistically novel transfer oxygenative cycloaddition process (Scheme 58C).

**Scheme 59:**
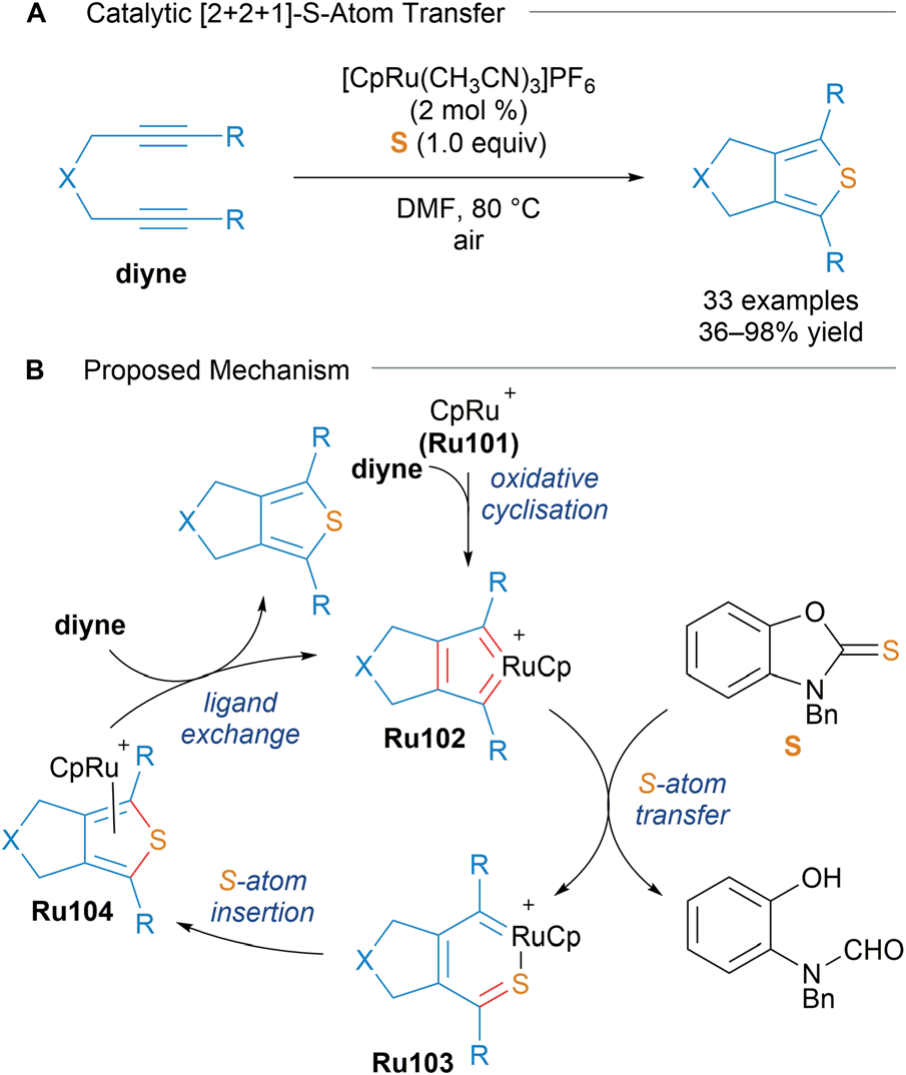
Sulfur atom transfer catalytic [2 + 2 + 1] cycloaddition for the synthesis of thiophenes.

The same group also realised an analogous transformation for the synthesis of fused thiophenes using benzoxazole-2-thione S as a sulphur atom donor in 2016 (Scheme 59).^100^ Treatment of the previously reported ruthenacyclopentatriene with a thione and AgPF_6_ (1.1 equiv.) led to the formation of thiophenes, suggesting its involvement in this atom-transfer [2 + 2 + 1] cycloaddition.

**Scheme 60:**
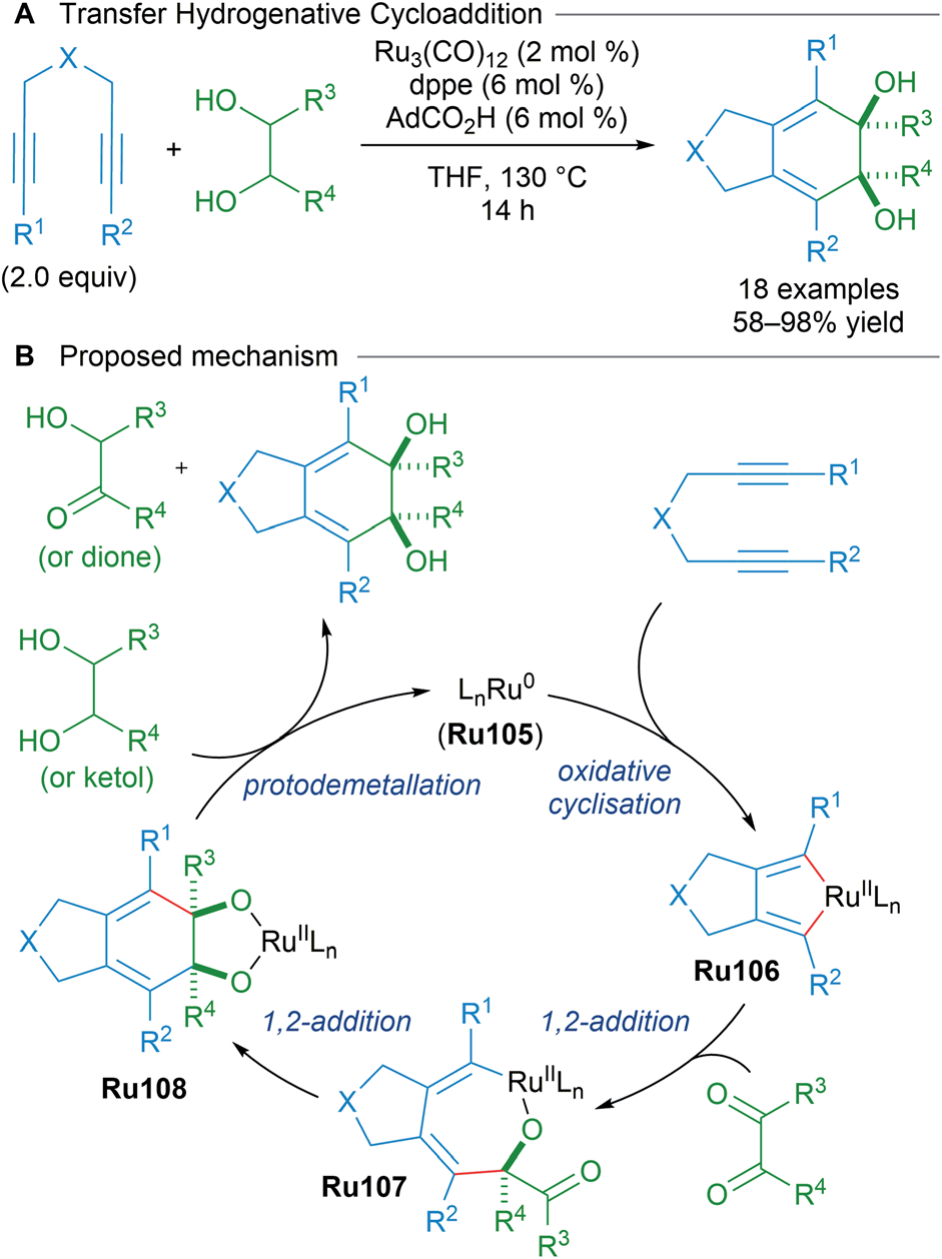
Krische's transfer hydrogenative cycloaddition.

As part of a longstanding program in expanding transition metal-catalysed reductive couplings of carbonyl compounds, Krische reported the likely generation of ruthenacyclopentadienes from 1,6-diynes through transfer hydrogenative coupling (Scheme 60).^101^ The data corroborate a mechanism in which Ru(0)-mediated oxidative coupling of a 1,6-diyne is followed by successive carbonyl addition between the resulting ruthenacyclopentadiene Ru106 and a transient dione. The dione can be generated from dehydrogenation of the diol using the 1,6-diyne as an hydrogen acceptor. An analogous strategy was later applied to type II polyketide synthesis.^102^

**Scheme 61:**
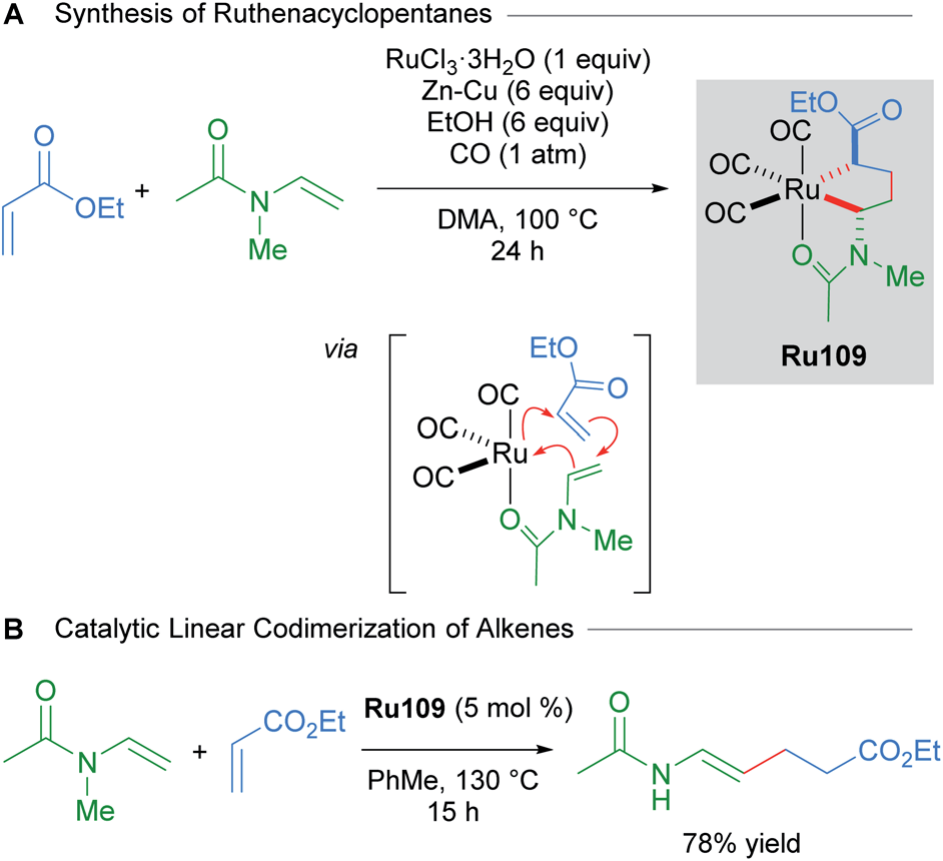
Linear codimerisation of alkenes by ruthenacyclopentane complexes.

Various saturated ruthenacycles have also been synthesised in recent years and applied towards catalytic bond formation. In 2017, Ura reported that ruthenacyclopentanes can be synthesised by the reaction of RuCl_3_·*n*H_2_O with Zn–Cu, *N*-vinylacetamides and electron-deficient alkenes (*e.g.* ethyl acrylate, dimethyl fumarate, and dimethyl maleate) under a CO atmosphere (Scheme 61A).^103^ The three CO ligands were found to coordinate in a *fac* fashion, with additional stabilisation provided by the coordination of the acetamide oxygen atom. The resulting complexes Ru109 were air- and moisture-stable, and were even amenable to purification by silica gel chromatography under ambient conditions. Alternatively, Ru109 could also be synthesised from [RuCl_2_(CO)_3_]_2_. The linear codimerisation of alkenes catalysed by complexes Ru109 proceeded in good yields (Scheme 61B). The authors suggest that a metallacycle mechanism for alkene codimerisation that proceeds through β-hydride elimination, followed by reductive elimination is likely, although the alternative possibilities have not been ruled out. This report represents a rare example of an exceptionally stable and isolable ruthenacyclopentane used in a catalytic bond-forming application.

**Scheme 62:**
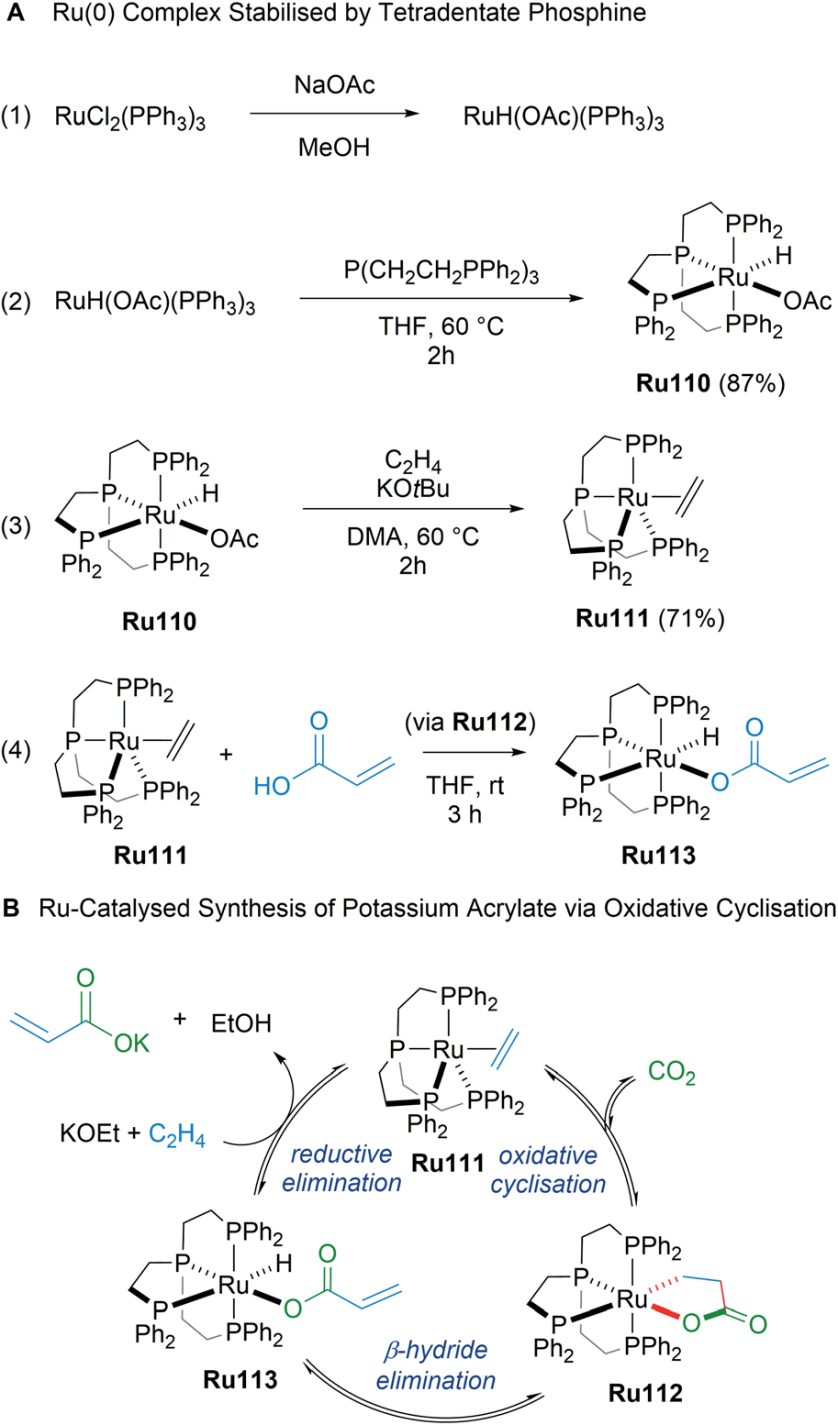
Ru-catalyzed synthesis of potassium acrylate *via* oxidative cyclisation.

In 2019, Iwasawa reported the synthesis of ruthenalactones from the oxidative cyclisation of ethylene with CO_2_ in the presence of electron-rich Ru(0) complexes with tetradentate phosphines (Scheme 62A).^104^ Treatment of ruthenalactones with a strong potassium base led to the release of potassium acrylates, thereby transforming CO_2_ into a valuable commodity chemical. This was the first example of catalytic acrylate synthesis from ethylene and CO_2_ using a ruthenium-based catalyst; however, the authors noted that the TON of 6 was low compared to existing palladium or nickel catalysts. In 2020, the same authors reported a follow up mechanistic study of this process that uses the readily prepared ruthenalactone complex Ru111 for stoichiometric reactions.^105^ The proposed catalytic cycle proceeds through an initial oxidative cyclisation of ethylene and CO_2_ to afford ruthenalactone Ru112, followed by β-H elimination to form the hydrido acrylate complex Ru113. Lastly, reductive elimination with a strong base leads to ethylene-bound, zero valent ruthenium species Ru111 to start the cycle anew (Scheme 62B). An alternative mechanism wherein deprotonation of ruthenalactone Ru112 by strong base was also plausible, although this likely depends on the strength of the base.

**Scheme 63:**
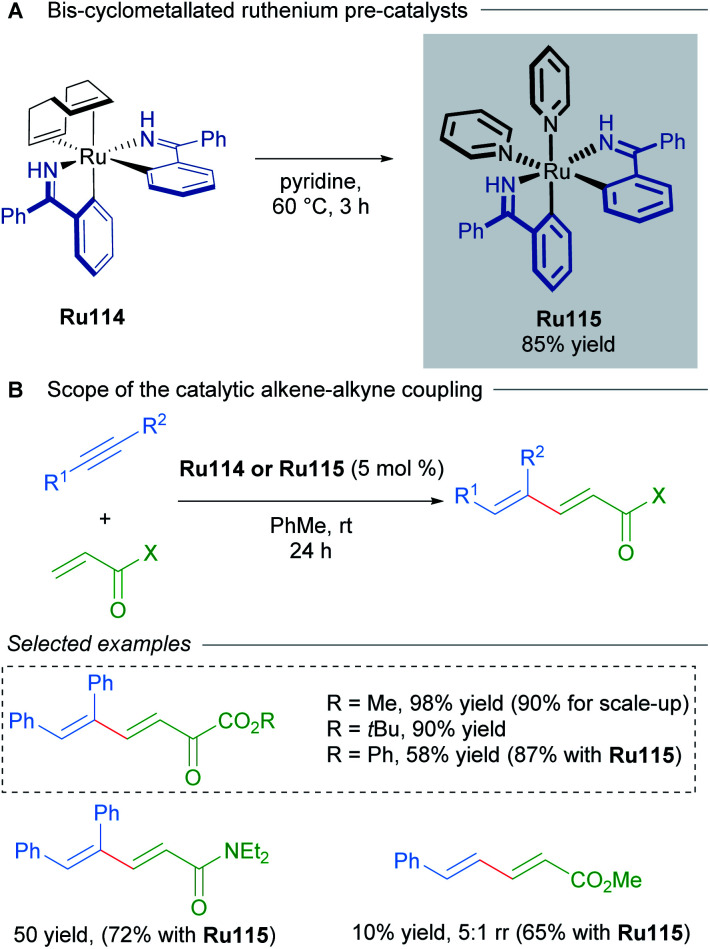
Bis-cyclometallated ruthenium complexes for alkyne–alkene coupling.

Bis-cyclometallated complexes have also been implicated in oxidative cyclisations. In 2014, Zhao reported the synthesis of bis-cyclometalled ruthenium complexes of benzophenone imine and used these as precatalysts for alkene–alkyne coupling (Scheme 63).^106^ Higher temperatures were required with non-cyclometallated precatalysts in this transformation. Optimisation revealed that pre-treating a previously reported complex^107^Ru114 at 80 °C leads to *in situ* formation of a bis-cyclometallated complex Ru115 (Scheme 63A), which catalyses the 1,3-diene synthesis at room temperature (Scheme 63B).

The wide variety of isolated ruthenacycles that have been applied to catalytic cycloaddition processes continues to grow, and it is expected that further developments in this exciting area will continue into the foreseeable future.

## 8. Conclusions and outlook

The use of cyclometallated ruthenium complexes in catalytic bond-forming processes has become increasingly widespread. In the last decade alone, significant advancements in C–H activation, *Z*-selective olefin metathesis, asymmetric cyclopropanations, cycloadditions, transfer hydrogenation, and chiral-at-metal catalysis have been realised with cycloruthenated complexes. In this review, we have provided an overview of these key research areas that take advantage of cycloruthenated complexes for organic synthesis. While the catalytic applications of these complexes are numerous, only a detailed understanding of the reactivity and behaviour of these complexes will enable their wider use in other chemical transformations. We hope the rich chemistry of cycloruthenated complexes presented here will inspire future developments in this field.

## Author contributions

M. T. F., P. D.-L., G. M., and A. Y. contributed equally to the writing of this manuscript. A. Y. conceived the idea and created the outline with equal contributions from M. T. F., P. D.-L., and G. M., with input and direction from I. L.

## Conflicts of interest

There are no conflicts to declare.

## Supplementary Material
